# Mind body medicine: a modern bio-psycho-social model forty-five years after Engel

**DOI:** 10.1186/s13030-023-00268-3

**Published:** 2023-03-30

**Authors:** Gregory Fricchione

**Affiliations:** 1 Benson-Henry Institute for Mind Body Medicine at Massachusetts General Hospital, Boston, USA; 2 Department of Psychiatry, Massachusetts General Hospital, Boston, USA; 3 Harvard Medical School, Boston, USA

“In simple scientific terms, the book---based on research my colleagues and I had published in medical journals—spelled out connections between mind and body that were reasonable and meaningful to Western scientists and their patients.”

Herbert Benson, Forward to the 25th Anniversary Edition of The Relaxation Response. 2000.

## Introduction

Over two years ago, the editors of this journal contributed an essay on the 13^th^ anniversary of the journal *BioPsychoSocial Medicine* [1]. The authors catalogued the multitude of approaches and modes involved in the biopsychosocial approach to illness and health first espoused over 45 years ago by George Engel [2]. Behavioral sciences, social sciences, neuroscience, stress physiology, epidemiology, psychoneuroendocrinology/immunology, gut-brain axis, psycho-cardiology and psycho-oncology are just a handful of areas that fall under the rubric of biopsychosocial medicine.

However, the problem with the Engelian approach to medicine is that physicians who have integrated it most into their work-life are not the time crunched doctors who see most of the patients with chronic stress-related non-communicable diseases (NCDs). By and large it has been psychiatrists and the health psychologists who have been most engaged with the biopsychosocial approach as an organizing principle.

Engel felt that the medical model sans adequate attention paid to the human psychosocial illness experience, which requires a scientifically rational approach to behavioral and psychosocial factors, is reductionistic and subject to mind-body dualism. This is because the internal and external conditions of the life one experiences are significant variables affecting timing and course of illness, patient and observer perception of illness, symptom maintenance beyond the physiological trigger, and the relationship between patient and doctor within which therapeutic response is often determined. With this in mind, Engel argued that a physician’s *“basic professional knowledge and skills must span the social, psychological, and biological, for his decisions and actions on the patient’s behalf involve all three.”* [2] (p 133).

Several years before Engel’s paper, the late Herbert Benson was investigating the physiological underpinnings of stress-related hypertension. This work led to his exploration of the benefits of meditation as an antidote to the overactive stress response system’s pathogenicity. His seminal book, *“The Relaxation Response”* published in 1975 summarized his research on the calming physiological state that can be achieved with a simple focused awareness breathing meditation [3].

In many ways this present review owes much to conversations I was fortunate to have with Dr. Benson over the years. His work and the work of other pioneers like Jon Kabat-Zinn (1990), with his writings on mindfulness as the cultivation of an open, non-judgmental awareness of one’s present state, greatly advanced the field of applied mind body medicine [4].

To be sure, gaining acceptance for modern mind body medicine with its the biopsychosocial approach from our colleagues in primary care medicine will be a multi-determined effort for the foreseeable future [5]. There are many pressures aligned against apportioning the time it takes to robustly consider the diverse components of a patient’s health or illness and to then integrate them in what is now sometimes called *whole person care* [6]. We have recently contributed our hope for the future of this effort in a perspective piece titled *“A New Era for Mind Body Medicine.”* [7].

## Modern mind body medicine and the bio-psycho-social approach

Perhaps the empirical basis of a non-dualistic mind body medicine will advance to the point that ignoring the benefits of a holistic approach will be considered outside the standard of care. The mind body approach includes relaxation response elicitation through meditation, mindful exercise like yoga and tai chi, cognitive skills training, positive psychology, problem solving and acceptance with commitment to eudemonic values and perhaps most importantly social support and pro-social behavior along with spiritual connectedness. These latter 3 aspects flow from secure attachment, the primary foundation for human wellbeing laid down by our mammalian evolution [8, 9]. All these resilience dimensions are enhanced by commitment to a healthy lifestyle that includes avoidance of smoking, excessive alcohol intake and recreational drug use, as well as exercise, sleep hygiene and a low glycemic diet. This curriculum for health is providing an evidence-informed syllabus essential for the self-care required to do effective primary, secondary and tertiary prevention. At the Benson-Henry Institute (BHI) for Mind Body Medicine at Massachusetts General Hospital, our 8-week composite Stress Management and Resiliency Training (SMART) Program includes these elements and we have published positive results in clinical and non-clinical populations [10–15].

However the SMART program is just one of many mind body interventions (MBIs) that can be used to promote biopsychosocial health. And certainly, more controlled prospective research will be needed to secure a place for mind body medicine alongside other accepted interventions.

But medicine is a human science and as such, evidence-based data must be combined with a heuristic evidence-informed narrative that makes sense to scientifically minded clinicians. Capturing the imagination of these doctors will ultimately promote the social reality that becomes operationalized in medical practice [16]. Here we suggest one such scientific narrative that makes the medical case for self-similar scalar influences that move up and down the human organism’s multiple biological, psychological and social systems. And to help visualize these relationships, we show figures used by scientists working at various systems levels to express the multi-level stress resilience that summates to the healthy and unhealthy phenotypes we see in medical practice.

## The stress resilience narrative

Stress is what the brain does to itself and the rest of the body when faced with a separation threat or challenge endangering one’s attachments. Stress can be said to come in three varieties--*normal, tolerable* and *toxic* threat stress depending on the degree of distressing uncertainty attendant to the threat.

When mammals evolved, another survival challenge joined the microbial threat and the predation fears shared by all vertebrates. Since the hallmark of the mammalian behavioral survival strategy is secure attachment to parental and social objects largely mediated by a part of the paralimbic cortex called the anterior cingulate cortex (ACC), a particular form of the acute stress response engendered by the amygdala became prominent in the life of mammals threatened with separation from social attachments [17]. In the course of evolution, the acute separation stress response became entwined with the acute inflammatory response, which also relies on the tripartite (hypothalamic-pituitary-adrenal axis, autonomic nervous system, innate immune response) stress response system for mediation and modulation [18].

Having an innate immune response at the ready when one’s brain perceives an acute situation as threatening to attachments provides a survival advantage, so much so that it will happen even in the setting of false positives. As a reflection of this evolutionary biology, the body’s defensive inflammatory response will respond not only to microbial threats but also to psychosocial threats. It is now suspected that, among signals appreciated by threatened cells are danger associated molecular patterns (DAMPs), non-pathogen associated molecular patterns (non-PAMPs) caused by psychosocial stress that join the pathogen associated molecular pattern (PAMP) microbial inflammatory triggers [19]. DAMPs play a key role in correcting an altered cellular physiological state, but when overactivated this defense can become harmful due to abnormal signal transduction [20].

As heirs to the evolutionary mammalian survival strategy, humans require secure attachment to sources of metabolic energy in food and drink, to sexual objects and to parental and social objects [21]. Evidence of this evolutionary trend in attachment need, comes from an elegant recent study showing that acute social isolation evokes midbrain craving responses similar to hunger induced food craving [22]. In other words, the brain processes the more recently evolved motivation to attach to social objects using elements of the same basic infrastructure that mediate the more primitive need for attachment to food.

In this study, subjects, forced to be isolated, crave social interactions in a similar way to hungry people craving food. Responses in the substantia nigra (SN)/ventral tegmentum (VTA) to food cues after fasting and to social isolation were correlated with craving [22]. Both food and social craving evoke responses in paralimbic regions in the medial prefrontal cortex (mPFC). Food craving evokes more selective responses in the ACC and (less reliably) in the insula and amygdala, whereas social craving evokes more selective responses in another connected paralimbic region, the orbitofrontal cortex (OFC). Fasting enhanced responses to food cues mostly in the nucleus accumbens (NAc), whereas isolation enhanced response to social cues mostly in the caudate. One can surmise from this experiment that evolutionary development of advanced attachments, as part of our mammalian and primate survival strategy, required appropriation of the ACC/OFC cortico-striato-thalamo-cortical (CSTC) brain reward/motivation circuitry.

In addition, as humans we attach to future objects in a prospective process of *constructive episodic simulation* sometimes poetically referred to as a *“memory of the future.”* [23–25]. In essence humans evolved to seek secure attachment; we are hardwired for this attachment solution to produce a biological, healing effect [26]. This is reflected in what might be called our attachment solution neurobiology. For example, we have evolved Von Economo spindle cells (ACC and frontopolar cortex) that integrate differentiated information in heteromodal cortex; mirror neurons that attach us in a Theory of Mind to others in our social milieu; the ACC-dorsolateral prefrontal cortex (DLPFC)-frontopolar central executive network in connection with emotion regulation and the brain reward and motivation circuitry that prompts our attachment behavior decisions; and the oxytocin receptor density network that affiliates us with our in-group [27]. A key to combating stress-related NCDs will entail meeting separation challenges with attachment solutions that maintain *“conditional independencies”* at every developmental scale [28].

When we face separation challenges to our attachments, our brains must adjust our physiologies to meet the environmental costs of these challenges while maintaining physiological stability in the face of these changes in a predictive process we call allostasis [29–31]. This task is more or less easily handled in the normal and tolerable stress zones but when faced with serious or chronic toxic threats, the energy required by the brain to keep physiologies from persisting outside the normative range, can become excessive leading to allostatic overload. If allostatic overload goes unchecked the risk of metabolic syndrome (MetS) will grow setting the stage for vulnerability to stress-related NCDs.

The relationship among the three stress zones can be described using a concept called hormesis [32–34]. In a recent review, Epel and colleagues (2020) define hormesis as *“a set of evolutionary well-preserved mechanisms of biological plasticity to survive and thrive when exposed to harsh circumstances and substances.”* [34]. Hormesis is therefore a form of stress resilience representing a biphasic effect of stress. Intermittent tolerable hormetic stress enhances physiological and psychological reserve capacity and can lead to stress inoculation or *stress-rejuvenescence* [34]. However, the overwhelming or persistent toxic threat-based form of distress can lead to *stress acceleration* and subsequently to illness. Toxic stress often lies outside the hormetic zone leading to a vulnerability to negative outcomes.

**Fig. 1:**
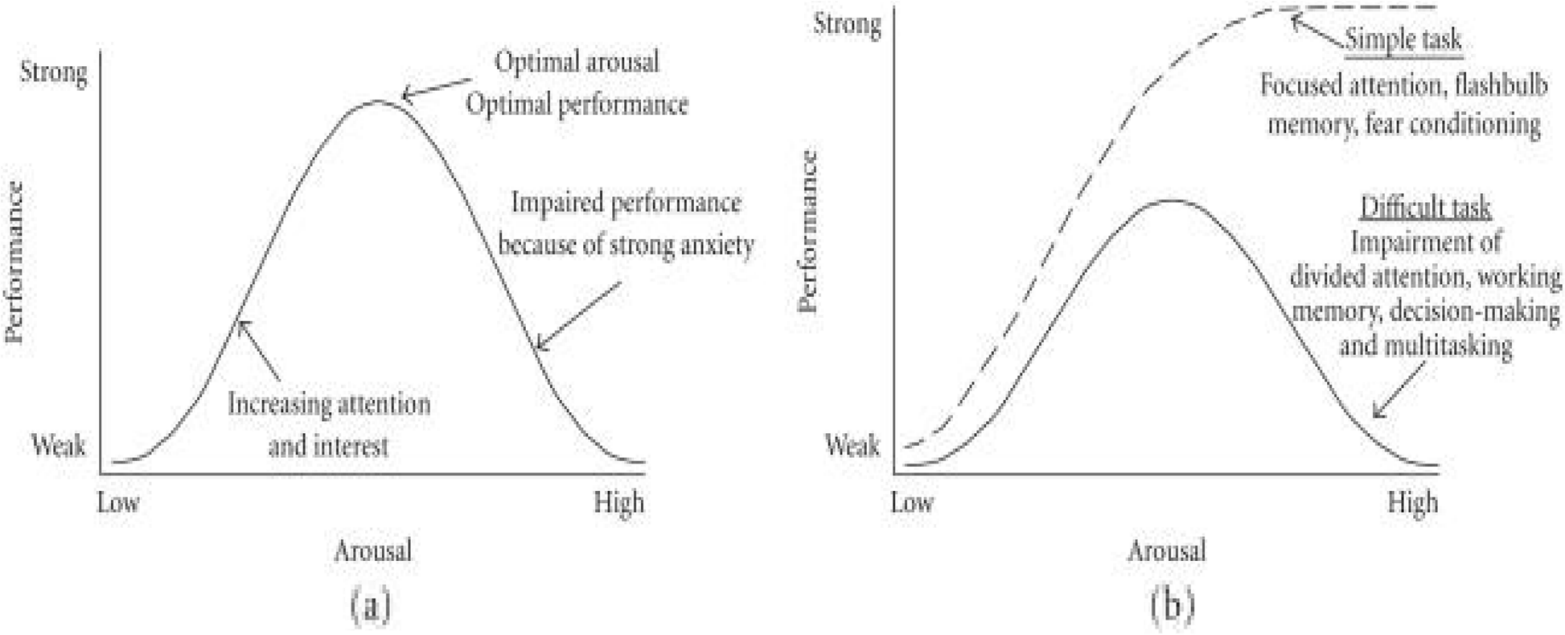
A version of the Yerkes Dodson Curve is depicted graphing performance on the y-axis and arousal or stress on the x-axis. The biphasic nature of the relationship is enhanced in the setting of task difficulty. From: Diamond et al, 2007, p. 3 (open access) [34]. *"Reprinted from Neural Plasticity 2007:60803.* Diamond DM, Campbell AM, Park CR, Halonen J, Zoladz PR. The temporal dynamics model of emotional memory processing: a synthesis on the neurobiological basis of stress-induced amnesia, flashbulb and traumatic memories, and the Yerkes-Dodson law, *with permission from Hindawi via STM guidelines"*

**Fig. 2:**
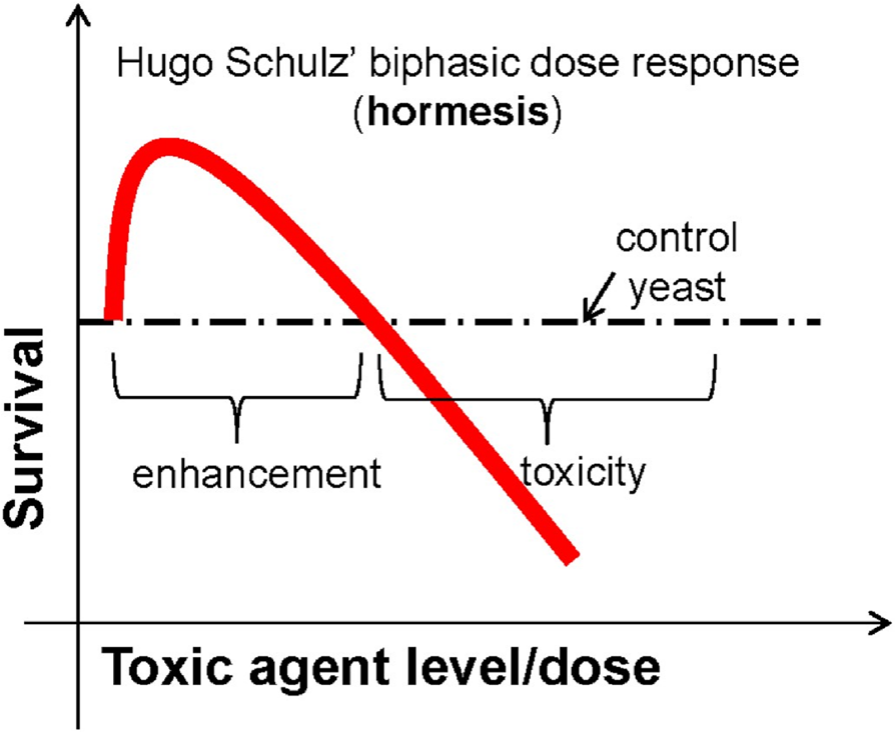
Hormesis Curve. From: Calabrese and Agathokleous, 2020, p 2, [35]. *"Reprinted from Environmental Research, 186,* 109559. Calabrese EJ, Agathokleous E.*,* Theodosius Dobzhansky's view on biology and evolution v.2.0: "Nothing in biology makes sense except in light of evolution and evolution's dependence on hormesis-mediated acquired resilience that optimizes biological performance and numerous diverse short- and longer-term protective strategies.” *P. 2,2020,* Apr 21* with permission from Elsevier via STM guidelines"*

The biphasic nature of the relationship between stress (*x*-axis) and health or performance or wellbeing (*y*-axis) appears to be a fundamental life principle and has been referred to as an inverted U-shaped, Bell-shaped, or biphasic curve [34]. This biphasic relationship is recognized in the Yerkes-Dodson Law where performance benefits from some stress but suffers when stress passes a certain level of personal tolerability particularly when dealing with complex or challenging learning situations, characterized by divided attention, multitasking, and working memory tasks [35–37]. It is also recognized in the Arndt-Schulz Curve, depicting the physiological relationship between the stress of the environment and bodily tissue functioning and the effect of certain drugs on receptor function [34, 35] (See Figs. 1 and 2).

In effect, brief intermittent, low dose stressors can induce positive biological responses promoting stress resilience [38]. After a disruption in homeostasis, hormetic stimuli may directly or indirectly in a restitutive, compensatory way provide anti-fragility [38]. The limited capacity for physiological adaptability in stressor response is usually ~ 30-60% higher than the maximum non-hormetic control response [33, 36].

Physical, biological/nutritional and psychosocial factors, which bring about hormesis are called hormetins. Stress dose, intensity and persistence combine with host characteristics to predict resilient physiological and psychological responses (e.g., post-traumatic growth) to hormetins as opposed to physiological and psychological damage and impairment (e.g., post-traumatic stress disorder [PTSD]). High dose and chronic exposure can overwhelm restitutive mechanisms leading to cellular oxidative damage, apoptosis, premature aging and death.

**Fig. 3:**
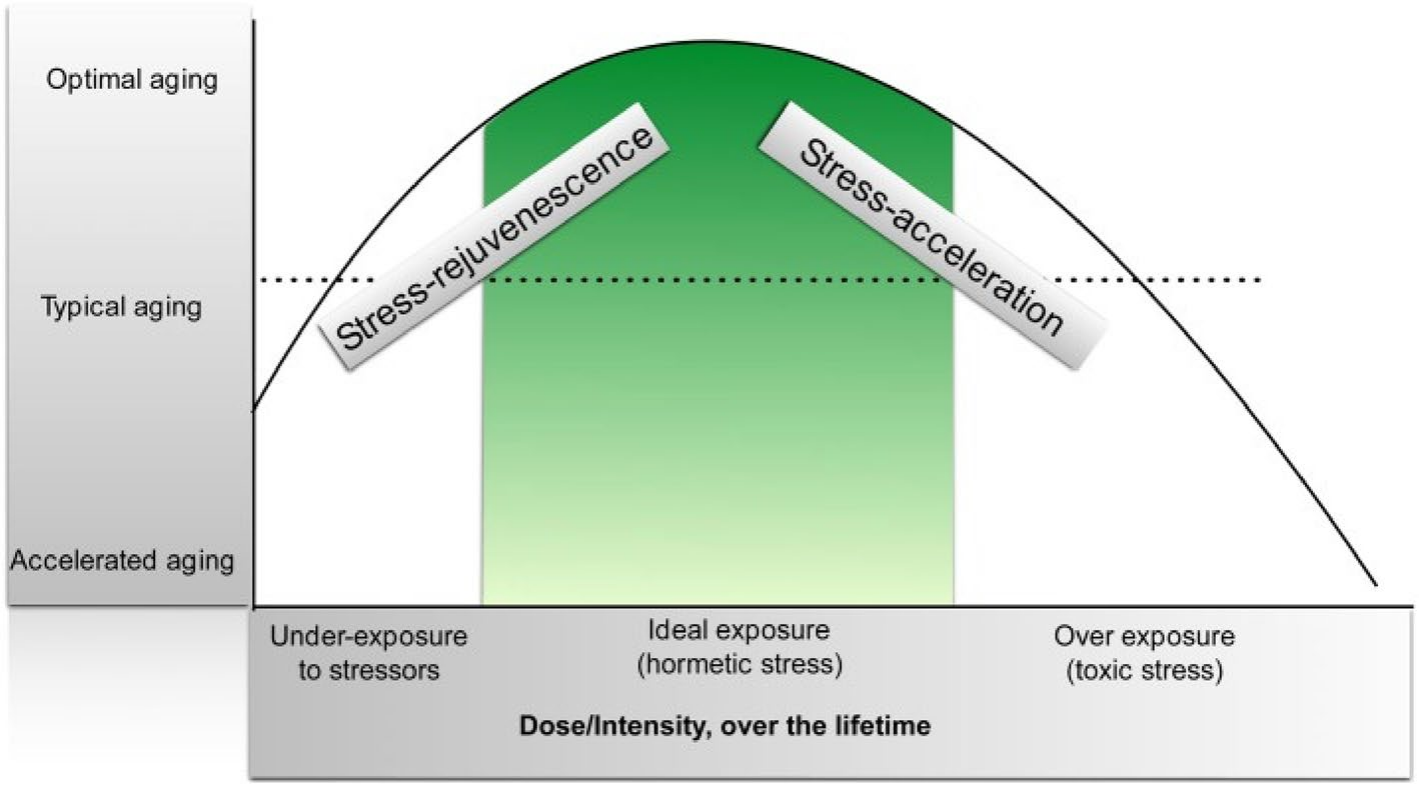
An Inverted U shaped hormesis curve depicting the longitudinal relationship of aging to the dose and intensity of stress over a lifetime. Epel, 2020, p.3, [34, 37]. *"Reprinted from Ageing Research Reviews, 63:101167.* Epel, E. The geroscience agenda: Toxic stress, hormetic stress, and the rate of aging. *P. 3, 2020,* Sep 28 *with permission from the author and from Elsevier via STM guidelines"*

In terms of stressors, under-exposure can lead to lack of development of stress buffering resources, and poor ability to quickly recover from stressors. Ideal exposure to sufficient numbers of manageable challenges throughout life support the development of emotion regulation capacity, cognitive growth, and coping skills, as well as to the cultivation of supportive social networks [34]. This kind of optimal exposure can give rise to hormetic functioning that is enhanced compared to baseline [34, 37] (See Fig. 3). Overexposure to stress without sufficient resources (*toxic stress*) to buffer against it can lead to maladaptive neural pathways in the connectome with an overresponse to stress and poor emotion regulation followed by depression and other NCDs along with stress-related acceleration of aging.

## The mind body medicine stress to resilience ratio

**Fig. 4:**
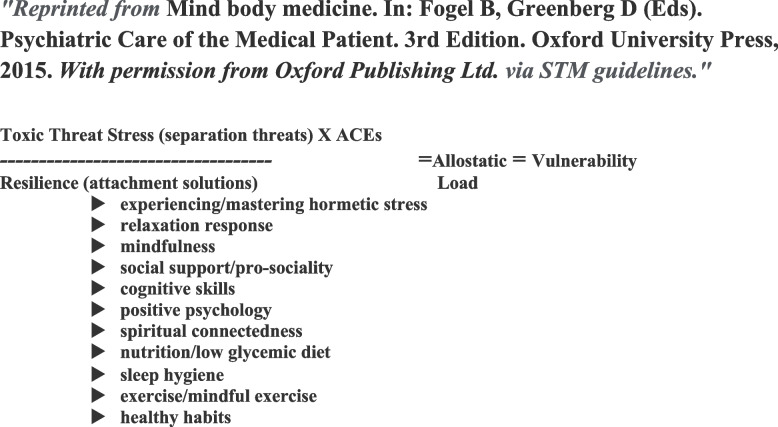
Mind Body Medicine Stress to Resilience Ratio – Adapted from: Fricchione, 2015 [39]. *"Reprinted from* Mind body medicine. In: Fogel B, Greenberg D (Eds). Psychiatric Care of the Medical Patient. 3rd Edition. Oxford University Press, 2015. *With permission from Oxford Publishing Ltd. via STM guidelines"*

One can think of overexposure to stress without sufficient resources (*toxic stress*) in terms of what might be called the mind body medicine ratio of stress to resilience [39] (See Fig. 4). This ratio can provide a rough estimate of allostatic load, a proxy measure for vulnerability to MetS and subsequent stress-related NCDs.

Building on the earlier work of social psychologist George Albee (1982), this ratio provides a heuristic approach to stress-related illness vulnerability [40]. This might be called an Illness Index or the propensity to illness. Flip the numerator to resilience and the denominator to stress and the quotient changes to propensity to health—a Health Index. For example, if one studies women with systemic lupus erythematosus, when you populate the variables in the equation and you stratify the groups into low social support leading to a decrease in the denominator, the propensity to a lupus flare will go up [41, 42]. There are many examples of this relationship in the literature [43–46]. A classic article by Ruberman and colleagues (1984) looked at over 2000 men status-post myocardial infarction (MI) during a 3-year study and characterized them according to life stress and social support and compared each group with a normal sample of post-MI subjects. Mortality over 3 years was significantly increased in both the high life stress and low social support groups. When high life stress was accompanied by low social support there was a concomitant very large mortality increase [47]. It is of interest that intensive cardiac rehabilitation programs designed to combine mind body approaches that lower stress and enhance social support and resilience together with traditional exercise prescription and nutritional advice show better outcomes than traditional cardiac rehabilitation and treatment as usual [48, 49].

Advanced imaging tools provide objective measurement of brain activation in areas like the amygdala impacted by psychosocial stress and threat signals. Resting state amygdalar metabolic activity (AmygA) can be measured using ^18^F- fluorodeoxyglucose positron emission tomography/computed tomography (PET/CT) [50]. Measured AmygA correlates with perceived stress in human subjects [50, 51]. It is upregulated in chronic stress conditions like PTSD [52, 53]. The AmygA measure is considered to be fairly stable over time with only a 2% median 3 month change in a clinically stable population [54].

Recent ^18^F-PET/CT research provides conceptual support for the mind body stress to resilience ratio. Tawakol and colleagues (2017) looked at PET/CT scans in a series of subjects who had ruled out for a cancer diagnosis and found a significant correlation among heightened psychosocial stress-related AmygA and bone marrow activation resulting in myelopoeisis and downstream arterial inflammation [50]. The authors propose that higher AmygA associates with increased cardiovascular disease (CVD) risk through this cascade involving heightened arterial inflammation (ArtI) as an endpoint signifying increasing risk for major adverse cardiovascular disease events (MACE) [50, 55]. This line of research offers a window onto the biopsychosocial determinants of health and illness. Chronic socioeconomic or environmental stress associates with heightened stress-related AmygA and the aforementioned higher risk of MACE.

We may surmise that when the numerator in the mind body stress to resilience ratio is high, the vulnerability to cardiac illness is raised. The stress numerator can be attributed to separation challenges and threats and indeed it has been shown that separation anxiety on the Relationship Scale Questionnaire is positively associated with amygdala responsiveness to negative faces and amygdala volume on fMRI [56].

In further research, the Tawakol team went on to ask whether, among individuals exposed to such stressors, increased neurobiological resilience (NBResilience), defined as lower AmygA despite stress exposure, reduces MACE risk. They also asked if this potential positive effect of resilience is secondary to decreases in bone marrow activity and arterial inflammation (ArtI) [55].

To test these hypotheses, 254 subjects underwent PET/CT and AmygA, bone marrow activity, and ArtI were evaluated. Known MACE-associated chronic socioeconomic and environmental stressors such as noise exposure, neighborhood median household income, and crime rate were assessed. Elevated stress exposure was then quantified and MACE within 5 years of imaging was tracked.

Of the 254 individuals studied, 166 met criteria for at least one chronic stressor. Among these subjects, 12 had MACE experiences and within this group, higher AmygA in the setting of the stressor was postulated to reflect lower resilience although individual resilience variables were not studied. Higher AmygA in the setting of the stressor significantly correlated with higher bone marrow activation, ArtI, and MACE risk. Mediation analysis suggested that the NBResilience (AmygA <1 SD above the mean)-associated 50% decrease in MACE risk was significantly mediated by decreased ArtI.

These data raise the possibility that enhancing NBResilience may decrease the burden of cardiovascular disease opening up a potential therapeutic niche for mind body approaches that may enhance NBResilience [55]. Such an effect may explain the tertiary prevention benefits of intensive cardiac rehabilitation, which are enhanced by adjunctive stress management and resilience training.

In another study, AmygA and adipose tissue volumes were measured, and serial blood assessments for diabetes mellitus (DM) were obtained in 232 subjects who underwent combined FDG-PET/CT imaging. Higher baseline AmygA predicted subsequent, new-onset DM, independently of adiposity and other DM risk factors. Furthermore, higher adiposity increased DM risk only in the presence of higher AmygA. In a separate cross-sectional cohort, higher AmygA associated with higher insulin resistance. Therefore, this study suggests that activity in the stress-responsive amygdala may predict the onset of Type II DM [57].

This research coincides with research exploring the relationship of the important ACC/ OFC CSTC circuit, insulin resistance and major depression, another burdensome NCD [58]. Central nervous system insulin resistance is associated with truncal adiposity, a hallmark of the MetS and a source of inflammation [59]. It turns out that the ventral striatum (VS) is particularly sensitive to insulin. And in a PET study, insulin increases glucose metabolism in the VS except in depression [60]. The ACC circuit, which connects to the VS, is central to the benefits of secure base social attachment and the developmental capacity to make attachment solution decisions that embellish mPFC emotion regulation through diminution of AmygA [61].

Tawakol and colleagues (2021) have also recently studied the Takotsubo syndrome (TTS), a heart failure syndrome triggered by stress and typified on scan by a left ventricular “takotsubo” pot configuration. The researchers hypothesized that the ratio of AmygA, reflecting fear conditioning overactivation, to mPFC cortical activation (mPFC A), reflecting emotion regulation capacity, would predict development of the TTS; those with the highest Amyg A to mPFC A would develop the syndrome earliest [62]. Put another way, they suggested that this ratio reflects an interaction between stress-associated neural regions (amygdala) that promote vs. those that attenuate the stress response (mPFC) [63]. Study findings were consistent with this hypothesis in that heightened AmygA and/or lowered regulatory activity in the mPFC accentuated the risk for stress-related TTS and its adverse physiological consequences.

Additional brain regions such as the insular^.^ cortex and hippocampus, known to participate in stress regulation and to have altered connectivity in individuals with prior TTS, were not evaluated in this study. Thus, while the ratio of Amyg A to mPFC A, which includes the ACC, appears to play a role in the risk for developing TTS, additional study will be needed to uncover the roles of other important brain areas in stress-related disease development.

Much has recently been proposed regarding the importance of so-called *“inflammaging”* for premature aging [64]. Can aging join NCD vulnerability as a related quotient of the stress divided by resilience allostatic load ratio? Another interesting study suggests that this may be the case [65]. Harvaneck et al (2021) examined whether resilience factors can buffer against the effects of cumulative stress on biological age acceleration. In order to do so they made use of recent advances in the use of so-called *“epigenetic clocks”* [66]. These clocks use DNA methylation-based profiles to accurately determine biological aging at the level of cells, tissues and individual organisms [67]. The authors selected a version of an epigenetic clock called the GrimAge Acceleration [68]. It is defined as the residual of the regression of GrimAge to chronological age; when this score is positive, biological age is considered greater than chronological age. 'GrimAge' is unique in that it was trained on time-to-death data and has outperformed its predecessors in predicting both morbidity and mortality [69].

This particular research was designed to study the impact of cumulative stress evaluated with the Cumulative Adversity Inventory (CAI). The CAI score was associated with accelerated GrimAge and its physiological consequences on processes like adrenal sensitivity and insulin resistance, as well as psychological and biological resilience effects on accelerated aging in a community sample of 444 subjects [65]. Results also showed that psychological resilience reflected by greater emotion regulation, measured with the Difficulties with Emotion Regulation Scale, was associated with less stress-related age acceleration. Stronger self-control, measured with the Self-Control Survey-Brief, was associated on mediation analysis with prevention of increased GrimAge scores and greater self-control buffered against insulin resistance.

To summarize, poor emotion regulation and poor self-control in relation to cumulative stress was predictive of accelerated biological age even after adjusting for multiple covariates (sex, race, BMI, smoking, alcohol use, income, marital status, and education) in a healthy young to middle age community sample. Furthermore, the relationship between cumulative stress and accelerated biological aging appears to be moderated by resilience in the form of capacity for emotion regulation and self-control. In other words, the Mind Body Medicine Stress to Resilience ratio suggests a way to measure the propensity to accelerated aging.

In a preliminary study using a different epigenetic DNA methylation clock, Pavanello et al (2019) have shown that relaxation response (RR)-based meditation done for 60 days can decelerate aging in a healthy sample as measured by a leukocyte DNAm AGE algorithm [70]. While acknowledging the need for larger more controlled studies, the authors concluded that: *“The analysis of the epigenetic clock therefore may represent an accurate tool to measure the effectiveness of lifestyle-based interventions for the prevention of age-related diseases.” p.10* [70].

A key feature of emotion regulation is the process of cognitive reappraisal. By altering one’s appraisal of the meaning of sensory experience, emotional responding can be modulated. The reappraisal process can be strenuous and is mediated in certain mPFC regions that also promote visceral control and regulation of the immune system. In a study of 157 healthy community volunteers, greater reappraisal-related engagement of the dorsal anterior cingulate cortex (dACC), also known as the mid-cingulate cortex (MCC), was associated with greater preclinical atherosclerosis measured with carotid artery intima-media thickness and inter-adventitial diameter, and with higher IL-6 levels, which were found to mediate the association between dACC engagement and preclinical atherosclerosis [71]. This suggests that strenuous attempts to regulate negative affect maintained by heightened Amyg A through reappraisal may contribute to MACE risk via a matrix involving mPFC activity, particularly MCC activity, and systemic inflammation.

This raises the question whether amygdala-driven bone marrow activation, in the cumulative toxic stress situation wherein emotion regulation is strained, requires compensatory mPFC activation levels of such magnitude that it drives pathogenic innate immune overactivation when coupled with the direct sympathetically driven inflammatory effects of AmygA.

Part of the complexity of the mPFC system involves contrasting effects on the autonomic nervous system (ANS) associated with the dorsal regions (anterior MCC and pregenual ACC) vs. the ventral portions (ventromedial PFC and subgenual ACC) [72]. This appears to reflect the fact that the dorsal mPFC mediates pro-threat, high sympathetic nervous system (SNS) output states that represent intolerance of uncertainty when unfamiliar, potentially dangerous anomalies present themselves. The result is a command to subcortical areas like the peri-aqueductal grey (PAG), locus coeruleus and the ascending reticular activating system (ARAS) to promote high amplitude and high frequency stress-based physiological responding with attendant metabolic wear and tear. On the other hand, the ventral mPFC regions are charged with anti-threat stress and pro-parasympathetic nervous system (PNS) response elements that provide reassuring emotion regulation. Provisionally, it might be concluded that this seeming mPFC functional bias may describe a marker for stress-related NCD risk.

However more recent research complicates the picture. It appears that overactivation of the primate ventral subgenual ACC (Brodmann Area BA25) accentuates stress-related cardiovascular and neurobehavioral responses to threat [73]. This raises questions about the above hypothesis that ventral portions of the ACC support parasympathetic functioning and emotion regulation. But the differentiated BA25 may hold the key to this confusion. BA25 is the major mPFC output area to the subcortical structures involved in aversive threat response [74]. This area is differentiated into a caudodorsal portion that responds to aversive stimuli with stress response sympathetic activation and a rostroventral portion that along with BA14 (OFC) is stimulated by appetitive signals [75]. The subgenual ACC is overseen by the dorsolateral PFC (dlPFC- BA9, 46) and the frontopolar cortex (BA10) and BA32, which has rostroventral as well as dorsocaudal ACC zones [74, 76]. These areas, which may serve as the reservoirs of our stress resilience, dampen BA25’s AMYG A effects theoretically reducing stress-related illness vulnerability [74].

It remains to be seen whether, with more granular imaging or optogenetic techniques, this complex visceral control matrix can be better delineated and, in the process, offer us a better understanding of the AmygA stress numerator/ mPFC A denominator mind body ratio.

Nevertheless, we may surmise that mind body interventions (MBIs) that include cognitive skills training might diminish NCD risk by modulating the intensity of cognitive reappraisal demands through effects on areas jointly involved in emotion and inflammatory regulation like the dlPFC, and parts of the mPFC, particularly the dACC (MCC), and the amygdala [71, 74].

When these findings are paired with the PET/CT findings described above, one can hypothesize that AmygA can be associated with cumulative stress and constitute the numerator while mPFC A can be associated with emotion regulation-based resilience and constitute the denominator in the Mind Body Medicine stress to resilience ratio. A high quotient may reflect *inflammaging* based on excess bone marrow activation leading to myelopoiesis and downstream arterial inflammation. This high stress to resilience ratio would predict accelerated biological aging as well as MetS with downstream NCD risk. Future studies employing epigenetic DNAm clocks may be able to elucidate this relationship.

If we conceptualize this same ratio in terms of the Yerkes-Dodson and Arndt-Schulz inverted U-shaped curves, we might say that as long as Amyg A is balanced by robust mPFC A, an individual will remain in the hormetic zone of normal to tolerable stress and enjoy so-called stress rejuvenescence with its attendant enhancement in health and performance [34]. However, in the context of cumulative toxic separation threat stress, when Amyg A is overwhelming and/or persistent, it is not uncommon for the ratio to become imbalanced with excessive amygdalar tone unbuffered by sufficient and effective mPFC emotion regulation capacity.

It is known that greater chronic stress and greater cumulative adversity measured with the CAI are associated with smaller volume in the resilience enhancing mPFC, ACC and insular cortex regions [77]. These areas are central to emotion regulation but also to active inference-based attachment solution decision-making [78].

Such a cumulative stress and low resilience situation ushers in the downward phase of the biphasic curve and represents the kind of energy sink that accompanies allostatic overload and the downward slope toward accelerated aging, exhaustion, burnout, cellular oxidative stress and eventual MetS and stress related NCDs.

## The biophysiological substratum

There is a biophysiological substratum that supports this biopsychosocial model. Mitochondrial stress responses are modeled according to the same bi-phasic inverted U-shaped curve encountered earlier with regard to performance (Yerkes- Dodson) and hormesis (Arndt-Schulz). Each cell in our body contains hundreds to thousands of mitochondria depending on tissue energetic needs.

In an illuminating book, the physicist Geoffrey West provides a primer on the importance of scale and power laws for human biology [79]. Organisms are subject to an energy power law that states, for example, that for every four orders of magnitude increase in mass (# of cells each with 100s to 1000s of mitochondria) on the *x-*axis, the increase in metabolic energy rate required along the *y*-axis is only three orders of magnitude. The result is a non-linear (sub-linear) slope of ¾ according to *Kleiber’s law* [79]. This is the basis of an important economy of scale. The larger an organism is, the less energy mitochondria need to produce per cell per second for its tissue. When the size of an organism is doubled, it doesn’t need 100% more energy to continue to grow during development; it only needs 75% more. This allows mitochondria to work less hard in producing enough energy for growth. This scenario predominates during growth and development though eventually as mature maintenance energy costs, which increase linearly with an organism’s mass, mount, there is less and less energy available to create new tissue and growth ceases, giving way to aging [79, 80].

Researchers in biology and information theory often employ the metaphor of *‘the nest’* to describe the lines of bidirectional influence between upper and lower scales. Terms like *‘nested hierarchy’* or ‘*nested set’* are often used and these nested hierarchies allow us to conceptualize contemporaneous differentiation depicted vertically and synthesis depicted spatially. The Russian Doll metaphor is a well-known meme that encapsulates the idea of separate yet attached cups of varying sizes that fit together in a whole.

In terms of the nested hierarchy, there are multiple layers atop the mitochondrial biophysiological substratum that assume a fractal pattern, eventuating in self-similarity and a recursive scalar invariance [79]. The self-similarity of the inverted U -shaped curves that are used to explain functional behaviors in the hierarchical layers, reflects the scale-free invariant nature of these relationships.

And here is where the concept of vulnerability to illness and accelerated biological aging secondary to allostatic overload comes into focus, providing a scientific infrastructure for biopsychosocial mind body medicine. When the energy necessary for tissue maintenance increases in the face of neuromedical derangements and/or toxic psychosocial stress, it does so linearly.

As the organism grows it not only needs energy to make new cells, it needs it to maintain old ones. This means that the metabolic rate available to create new cells, is the quantity of metabolic energy produced by mitochondria left after that used for the maintenance of existing tissue [79]. As stated, the maintenance energy requirement expands in a linear fashion in line with mass, while the metabolic energy supply increases sub-linearly according to the ¾ power law [79, 80]. Consider the maintenance energy needs when an individual is under great levels of toxic stress producing allostatic overload. As maintenance energy requirements increase, the metabolic energy supply will be incapable of keeping up. This constitutes the kind of MetS deficit spending that will predispose to tissue damage compounded by reduced creation of new cells.

Mitochondria, as descendants of a unicellular bacterium endosymbiotically ensconced in each cell of the multi-cellular organism, respond to the stress they experience by sensing it, analyzing it and effecting a response a la any living organism [81]. Over evolutionary time and ongoing selection pressure, the process of mito-nuclear matching resulted in an intricate mito-nuclear relationship that is genetically integrated; the 2 genomes co-evolved to cross-regulate and support each other [82]. Undue stress will tend to disrupt this finely tuned collaboration and impair cellular functioning.

So just as we see the psychological Yerkes-Dodson Curve and the physiological Arndt-Schulz Curve of the human organism, we again see an inverted U-shaped bi-phasic curve emerge in the life experience of each of our mitochondria.

Indeed, we can speak of mito-hormesis and define it as a mitochondrial process wherein tolerable amounts of stress have the tendency to enhance the capacity of mitochondria to deal with the energy needs of future stress challenges through mitochondrial remodeling and the priming of anti-oxidant pathways [83]. This may frame the stress inoculation idea at a very basic level.

For example, tartrate-resistant acid phosphatase (TRAP)-1 protein inhibition in mitochondria occurs under stress conditions [83]. The result is reactive oxygen species (ROS) generation in amounts that cause retrograde stress signaling from mitochondria to nuclear DNA with induction of Forkhead Box (FOXO) mediated transcription of cell protection antioxidant genes. When all goes well, this produces feedback to mitochondria that activates a mitochondrial antioxidant defense and remodeling process to protect cells from future insults.

This mito-nuclear matching is an adaptive part of the bi-phasic response to stress at the mitochondrial level but it can give way to a maladaptive phase transition marked by inversion of the curve at the top when the energy demands of overwhelming and/or persistent separation stress drive the mitochondria out of the mito-hormetic zone and into pathogenic oxidative stress marked by mitochondrial fission and overproduction of ROS. Mitochondrial fission in the setting of toxic stress, can prompt the removal of hopelessly damaged mitochondria by facilitating apoptosis thus aiding in cellular quality control. The attachment solution of mitochondrial fusion on the other hand can mitigate stress via a process of complementation giving partially damaged mitochondria a fighting chance to regain health by mixing the best contents of the fused mitochondria [84]. In essence mitochondrial fusion is a resilience enhancing attachment solution the cell will use to expand its hormetic zone.

**Fig. 5:**
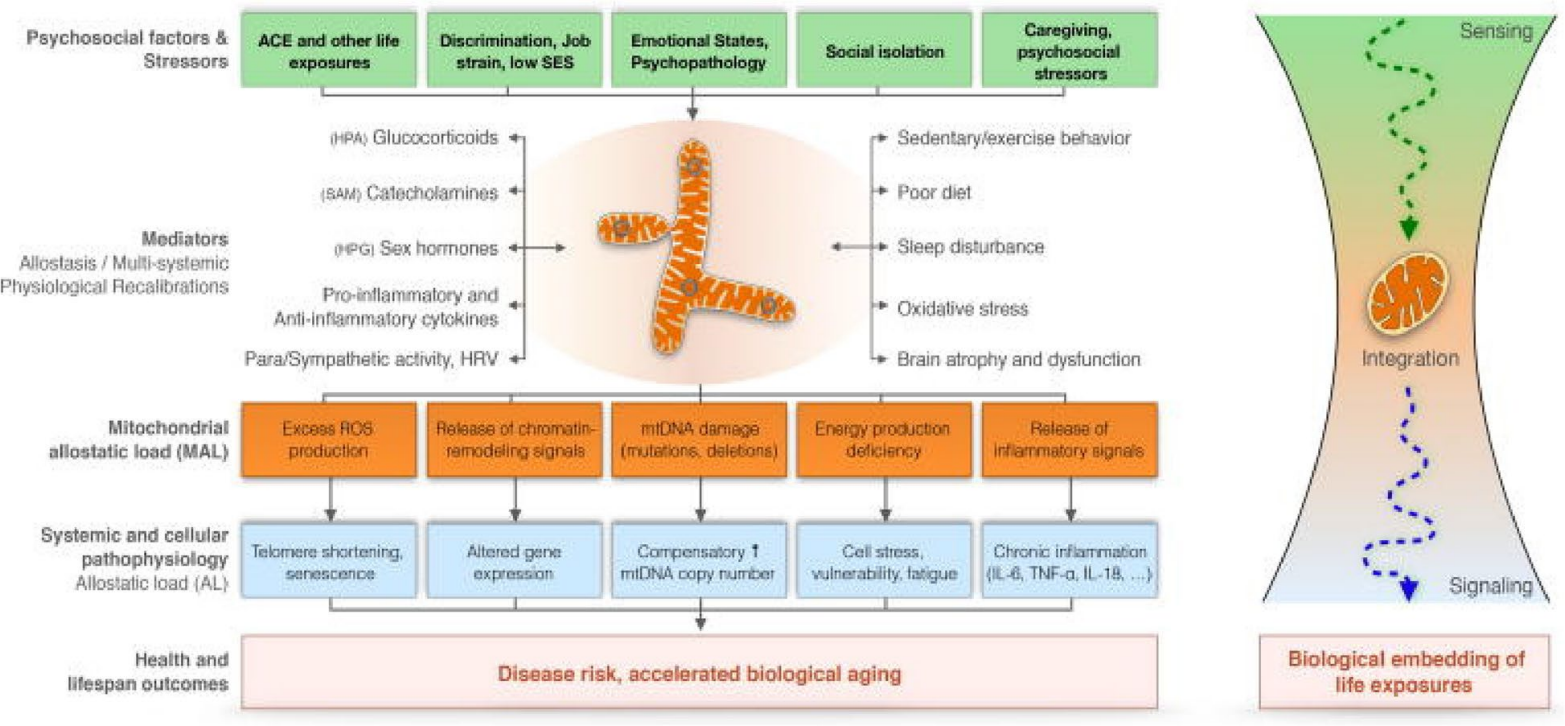
Each mitochondrion is a sensory-motor analyzer-effector that responds to sensory stimuli contextualized by psychosocial factors and other stressors that in turn engender a variety of mediators, which will differentially affect mitochondrial functioning. If stress is toxic to the organism’s attachments in the setting of a handicapped resilience state, mito-allostatic overload may ensue leading to a vulnerability to accelerated biological aging and NCD and viral pathophysiology. From: Picard and McEwen, 2018, p. 30, [85]. *"Reprinted from Psychosomatic Medicine,* Feb/Mar; 80(2)*.* Picard M, McEwen BS*.* Psychological Stress and Mitochondria: A Conceptual Framework. *P. 30, 2018, with permission from the author via STM Guidelines*

Resilience at the mitochondrial level can be measured by the duration mitochondria can sustain an adaptive response to the stressor before undergoing a decline in mitochondrial health below baseline. Therefore, a mitochondrion can be conceptualized as a living entity that must attend to its own environmental stressors in a biphasic way while brain and body spatiotemporally recapitulate this stress to resilience relationship at a macrosystem level [85] (See Fig. 5).

**Fig. 6:**
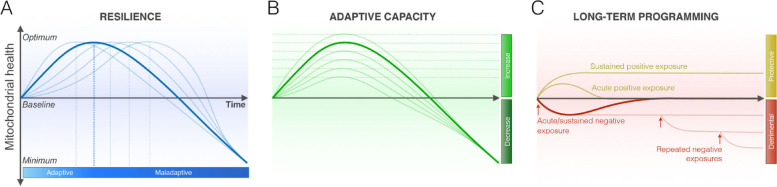
Mitochondrial Resilience and Adaptation. Developmental detrimental embedding may handicap mitochondrial resilience and adaptive capacity while protective embedding will enhance them. Persistent stress activation leads to mitochondrial allostatic load (MAL), which is then transmitted via molecular signals into cellular physiology changes resulting in overall allostatic load (AL). Energy ramifications of these micro-physiological states will be influential all the way up the line at the level of the organism’s macro-system health and social performance. Picard and McEwen, 2018, p. 31, [85]. *"Reprinted from Psychosomatic Medicine,* Feb/Mar; 80(2)*.* Picard M, McEwen BS*.* Psychological Stress and Mitochondria: A Conceptual Framework. *P. 31, 2018, with permission from the author via STM guidelines"*

Picard and McEwen (2018) depict this mitochondrial level effect using a series of inverted U-shaped curves with each curve in the series meant to reflect a cellular or physiological system with a different degree of resilience [85] (see Fig. 6). Certain stressors, particularly during sensitive developmental windows (separation-individuation and object constancy, social acceptance during latency, puberty and adolescence) when separation challenges are most problematic, can have long-term programming effects that establish lasting set points (detrimental or protective) for mitochondrial health. For example, adverse childhood experiences (ACEs) can be responsible for what is called detrimental embedding while social acceptance and secure attachment can lead to protective embedding. The former will sometimes handicap mitochondrial resilience and adaptive capacity while the latter will often enhance them. Energy ramifications of these micro-physiological states will be influential all the way up the line at the level of the organism’s macro-system health and social performance [86].

Of particular importance, given the key role played by inflammation in the pathogenic effects of an imbalanced stress to resilience ratio, is the relationship between mitochondrial wellbeing and macrophage/microglia functioning. Indeed, macrophages can be primed for apoptosis due to inducible nitric oxide licensing of the caspase 8 activation of a mitochondrial cell death pathway set in motion by IFN-γ as part of a hyperactive innate immune response [87].

Macrophages and microglia are immunosensors of the stress response [88]. They link up the innate immune response with the psychosocial stress response. Acute and chronic stressors induce a spectrum of alterations in macrophage-microglial function and immunophenotype. These alterations are mediated, in part, via stress hormones/transmitters including glucocorticoids (GCs) and norepinephrine (NE) along with the effects of cytokines and chemokines. The blood brain barrier (BBB) and the choroid plexus are areas of fenestrated endothelium in cerebrovascular capillary beds that serve as thoroughfares for signaling between macrophages in the periphery and microglial cells in the CNS [89–91].

As mentioned earlier, it is known that in all multicellular organisms, innate host responses to both microbial and sterile threats are initiated by so-called *‘danger responses.’* [92]. These are primitive systems that deliver cellular *‘danger signals’* when ‘*pattern recognition receptors*’ (PRR) come across activating molecules called *‘alarmins’*. Alarmins may be of pathogenic microbial origin (pathogen-associated molecular patterns or PAMPs) or of sterile endogenous origin (non-PAMP danger-associated molecular patterns or DAMPs). There are many infective and sterile alarmins and they all can result in an innate inflammatory response due to their shared capacity to activate PRRs. It is this overlap between PRR activation via both DAMPs and PAMPs that gives rise to the non-dualistic final common pathway we see with both sterile psychosocial and infective microbial innate immune responses [92].

Mitochondria, descendants of ancient anaerobic bacteria, are central to the link-up of the stress and inflammatory responses as reviewed above since they occupy the crossroads between sterile DAMPs and infective PAMPs at the cellular level [92]. Many mitochondrial proteins are considered alarmins. When responding to heightened cellular danger signals, the release of mitochondrial products from injured cells results in inflammatory stress responses.

This suggests that psychosocial stress can directly cause a non-PAMP DAMP microglial pro-inflammatory state that can indirectly lead to peripheral inflammation. Conversely a PAMP peripheral immune response as might occur in the microbiome can indirectly lead to neuroinflammation as well [92]. In both cases, the canonical pathway of the nod like receptor family pyrin domain containing 3 gene (NLRP3) inflammasome activation is a central factor [93].

This process is thought to involve two phases [87, 93]. The initial priming phase involves PRR signaling through toll-like receptor 4 (TLR4) recognized PAMPs such as lipopolysaccharides (LPS); or through signs of cell damage such as cell membrane or organelle membrane breaches, which can be sterile. In the case of LPS, the pro-inflammatory transcription factor NF-κB is activated, which drives transcription of IL-1β and NLRP3 mRNA, leading to translation of pro-IL-1β and inflammasome components in this first priming phase. In the second phase, another activating signal causes NLRP3 to form a complex, which recruits pro-caspase-1, which is then converted to the mature enzyme caspase-1. Caspase-1 then catalyzes the conversion of pro-IL-1β to mature pro-inflammatory cytokine IL-1β [19]. IL-1β can then start a cascade with the production of other pro-inflammatory cytokines and mediators, resulting in an amplified inflammatory response syndrome (IRS).

The immune system is well integrated with the stress response system in large part due to the intercession of NF-κB [94]. Catecholamines from SNS fibres increase NF-κB DNA binding in immune cells, including the macrophage, resulting in the IRS [94, 95]. In addition, the high mobility groupbox-1 (HMGB-1) *Alarmin* protein can trigger a DAMP that then signals cellular distress and stimulates an innate IRS. Indeed, macrophages release HMGB-1 as master regulators of innate immunity [96]. Once released into the extracellular space HMGB-1 initiates an IRS through effects at TLRs.

It is postulated that microglia release HMGB-1 in response to acute or chronic psychosocial stress [19]. Microglia have receptors for stress hormones and IL-1β. As mentioned above, it is possible in this way that psychosocial stress can cause neuroinflammation via a non-PAMP pathway. There are likely many other molecules (e.g., heat shock proteins) that signal in a similar manner.

When bearing in mind life-stress effects and their relation to psychosocial stress- related illness, a potential link to consider is a nucleic acid-sensing pathway connected to the immune response. Sterile DAMPs can be endogenous self-molecules like DNA materials that seep into the cytosol and become modified and thereby agonistic at PRRs or they can be unmodified highly concentrated self-molecules that come in contact with PRRs that are usually sequestered [97]. A protein named *cyclic-GMP-AMP synthase* (cGAS) is an innate immune system receptor which detects pathogenic DNA and alerts an innate immune adaptor protein, *stimulator of interferon genes* (STING), to mount an interferon (IFN) based response to protect the host [98]. Chronic low-grade inflammation can be activated by the cGAS-STING system as it senses cytosolic self-DNA in the form of mitochondrial DNA (mtDNA), which has slipped through mitochondrial pores due to inner and outer membrane permeabilization related to elevations in ROS potentially induced by toxic psychosocial stress [97, 98]. For example it is known that oxidatively stressed mitochondria can release mtDNA fragments through voltage dependent anion channel pores in the mitochondrial outer membrane [99]. In addition, when mitochondrial overproduction of ROS under conditions of stress leads to mitochondrial dysfunction, the mtDNA packaging transcription factor TFAM becomes depleted leading to leakage of mtDNA into the cytosol and activation of a cGAS-STING stimulated innate immune response [100, 101].

Stress-induced non-PAMP inflammation alone can be associated with a neuroinflammatory cytokine profile related to gene expression upregulation of NF-κB and type I IFN pathways [102]. This inflammation has been shown to be driven by the cGAS-STING pathway [98]. Excessive engagement of the cGAS–STING pathway in the brain (especially by microglia) can lead to neuroinflammation and neurodegeneration [98]. It is increasingly clear that COVID-19 pathogenesis owes much to cGAS-STING engagement in macrophages and endothelial cells raising questions as to how viral invasion and psychosocial stress conspire to afflict patients, including those with neuropsychiatric post-acute sequalae of COVID-19 [103]. Future targeting of the cGAS–STING pathway may afford therapeutic benefits in disorders related to psychosocial stress.

It should also be noted that, if this speculation that severe psychosocial threat stress, through its capacity to cause mitochondrial oxidative stress, can access the same cGAS-STING pathway that mediates the pathogenicity of non-self microbial threats, we have a new biological basis for the mind body unity of biopsychosocial medicine through neuroinflammation and the cGAS-STING pathway [104, 105].

Activation of the NF-κB that drives transcription of a large set of proinflammatory genes crucially depends on the activation of the integrative stress response (ISR) [106]. The ISR is an evolutionarily conserved intracellular signaling network used by cells and tissues to adapt the organism to environmental changes and thereby preserve health. In its latent state, NF-κB is anchored in the cytoplasm by its inhibitor IκB. Because IκB is a short-lived protein, it requires constant synthetic regeneration. The translation inhibition exerted by the ISR triggers NF-κB activation by lowering IκB’s steady-state concentration.

In addition, NF-κB activation in response to microbial PAMPs or oxidative stress related non-PAMPs is dependent on the ISR kinase, heme-regulated inhibitor (HRI), which in turn assembles downstream into inflammasome complexes [107]. The inflammasome then assembles the enzymatic machinery that degrades IκB, the stabilizing molecule for NF-κB. This disinhibits NF-κB and sets the stage for pro-inflammatory cytokine production. The inflammasome is therefore key to the innate ISR, a process that reduces overall energy depleting protein synthesis, while sparing necessary pro-inflammatory function.

In the setting of persistent or overwhelming stress, caused by diverse cellular conditions in both the lumen of the endoplasmic reticulum (ER) and the cytosol, the ISR in the ER is activated [106]. Gene expression analyses have revealed complex ISR-driven reprogramming of translation in the ER. In response to different conditions affecting protein homeostasis (proteostasis) such as protein folding defects, nutritional deficiencies, viral infections, and oxidative stress, the ISR attempts to maintain allostasis in the face of change by reprogramming gene expression. Abnormal ISR signaling can promote the pathogenesis of complex metabolic diseases, including stress-related NCDs.

Four specialized kinases (PERK, GCN2, PKR as well as HRI) respond to stressors with phosphorylation of a single serine on the eukaryotic translation initiation factor eIF2. This eIF2 phosphorylation inhibits eIF2’s guanine nucleotide exchange factor termed eIF2B. The result is a diminishment in energy depleting protein synthesis during the stressful environmental crisis. The ISR therefore minimizes the adverse effects of inflammation on glial cells [107].

The ISR is terminated by dephosphorylation of eIF2α. The molecule Sephin1 inhibits eIF2α dephosphorylation, thereby prolonging the protective ISR [107]. Chen et al (2019) studied the effectiveness of Sephin1 in protecting oligodendrocytes from allostatic overload and showed that Sephin1 prolonged eIF2α phosphorylation in stressed primary oligodendrocyte cultures [107].

Another molecule called the integrated stress response inhibitor (ISRIB) can reverse the effects of eIF2 phosphorylation [108]. It can thus restore translation by eIF2B dephosphorylation. In the mouse model, it has been shown ISRIB administration enhances cognition and minimizes cognitive decline due to brain injury [109].

**Fig. 7:**
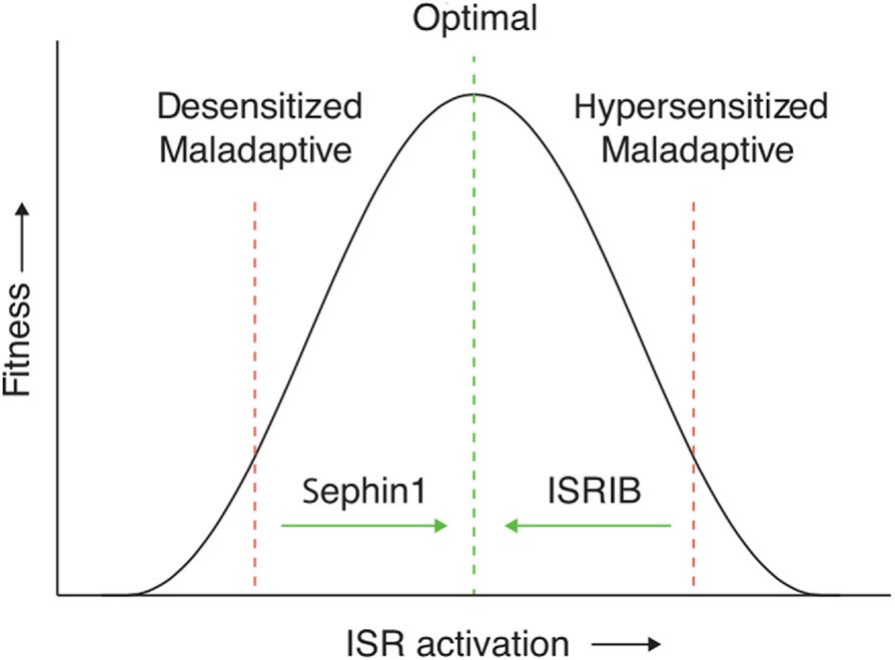
Model for proteostasis control by the ISR. Different pathologies may have distinct hormetic set points that relate to phenotypic fitness, such as cognition and behavior. As considered here, either reduced or increased ISR activation can be maladaptive. Therefore, depending on the disease or pathology and the optimal hormetic set point for a particular phenotype, activation of the ISR (e.g., with sephin1) or inhibition of the ISR (e.g., with ISRIB) would restore optimal homeostatic cell fitness. Costa-Mattioli and Walter, 2020, p. 7, [106]. *"Reprinted from Science. 2020 Apr 24; 368(6489).* Costa-Mattioli M, Walter P. The integrated stress response: From mechanism to disease. p. 7, 2018*, with permission from the American Association for the Advancement of Science via STM guidelines."*

In responding to PAMP as well as non-PAMP induced stress, cellular proteostasis mechanisms will attempt to preserve the hormetic zone vis a vis the ER and its translation function. The inverted U-shaped biphasic curve here will describe the relationship between fitness and the ISR. As the ISR increases, fitness improves up to an optimal point [106] (See Fig. 7). Either reduced and desensitized or increased and hypersensitized ISR activation can be maladaptive. When desensitized, Sephin 1 can advance fitness by promoting ISR activation. (See Fig. 7) When hypersensitized, ISRIB can promote optimal fitness by inhibiting the ISR.

While protein synthesis is decreased, there is simultaneous selective translation of transcripts that support adaptive stress responses [107]. By ratcheting down overall mRNA translation while activating the synthesis of a few proteins that initiate a transcriptional crisis plan, the ISR strives to maintain physiological homeostasis and proteostasis. However, if the stress becomes toxic and overwhelms the system, the ISR will trigger apoptosis to remove the damaged cell.

Simultaneously then, mitochondria, ER and innate immune cells are serving as immunosensors of alterations in mito-allostasis, proteostasis and allostasis that may push the organism outside the hormetic stress zone setting the stage for allostatic overload and subsequent inflammaging and illness. Because psychosocial instigation accesses the same pathways as microbial and metabolic instigators, there is increasing recognition that there is no separation, save perhaps for “dose effect” differences, between biological, psychological and social determinants of health and disease.

**Fig. 8:**
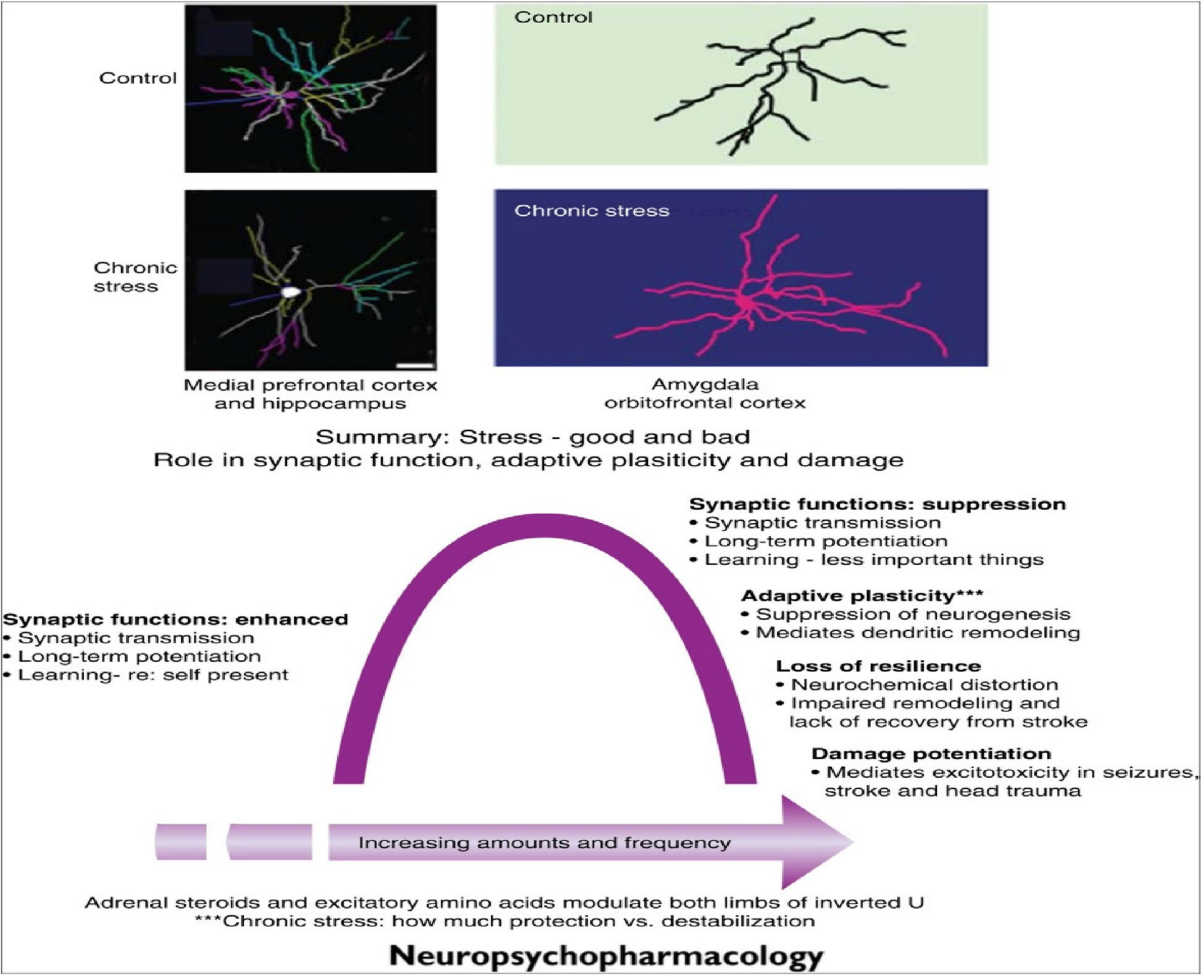
Neurons and Effects of Stress. Chronic stress in increasing amounts and frequency will cause a biphasic curve reflecting negative remodeling of dendrites and synaptic connections in many brain regions, including hippocampus, amygdala, medial prefrontal and orbitofrontal cortex (upper section). Such effects of acute and chronic stress operate in space and time in an inverted U-shaped manner (lower section). Acute stress, mediated by glucocorticoids, excitatory amino acids and other mediators can enhance excitability and promote memory over minutes to hours as long as the stressor is not overly intense; intense stress can have the opposite effect. Chronic stress causes neuronal remodeling as depicted in the upper section. This is usually largely reversible, as toxic stress subsides, promoting adaptation. Yet, if there is no reversal of the stress-induced changes in neuronal architecture, chronic stress-related NCDs have a better chance of emerging. From; McEwen et al, 2016, p. 13, [110]. *"Reprinted from Neuropsychopharmacology Reviews 2016; 41, 3–23.* McEwen BS, Nasca C, Gray JD. Stress Effects on Neuronal Structure: Hippocampus, Amygdala, and Prefrontal Cortex. *P. 13 with permission from Springer Nature via STM guidelines"*

One waystation with direct effect from mitohormesis and proteostasis will be at the level of the neuron [110] (See Fig. 8). Toxic stress (overwhelming and/or persistent) will precipitate a neuronal crisis reflected in negative remodeling of synaptic linkages and dendritic trees. This occurs in the hippocampus and amygdala and also in the mPFC including the ACC and OFC.

The pathway of neuronal information processing in the context of stress management involves mineralocorticoid receptor (MR)-dominated signaling cascades crucial for the brain’s analyzer-effector functioning [111]. The cortisol preferring MRs in the paralimbic and limbic brain control the initial phases of the stress response when circulating GC concentrations are low. These MR responses activate the circuits underlying mPFC (ACC/MCC/OFC) appraisal functions and a coping style that permits a move toward an explicit more deliberate emotion regulation rather than a more stimulus-bound *“if-then”* implicit emotion regulation style [111]. Then, when GC concentrations rise, the glucocorticoid receptor, (GR)-mediated actions are stimulated thereby dampening cellular stress reactions and allowing, through the use of active inference and Bayesian decision-making, the amalgamation of bottom-up emotion and top-down cognition that results, when all goes well, in selection of an appropriate motor response [78, 111]. At the same time GR-mediated responses promote behavioral adaptation as well as memory consolidation [111]. This sets the stage for the priming of these circuits for prospection with enhancement of threat preparedness. When re-encountering a similar threat, the GR-mediated consolidated memory provides a substrate for retrieval by a MR-mediated mechanism [111].

This “MR:GR balance” stress response adaptation hypothesis can be graphed with cortisol level on the *y*-axis and response time on the *x*-axis [111]. It turns out to be a biphasic inverted U-shaped curve with self-similarity to the Arndt-Schulz and the Yerkes Dodson curves as well as those of mito-hormesis and the ISR. As time of the response increases, cortisol rises. The convex non-linear rise on the left represents the bias toward the MR effects of appraisal and coping style (hormetic stress) that gives way at the peak cortisol levels to GR mediated actions and feedback inhibition with eventual reduction in GCs and a cessation of the acute stress response but with a memory of the stress experience for later access when needed. There are challenges to this hormetic story that emerge when this process becomes perseverative or when the stress has become toxic. When this happens, the amygdalar-driven HPA axis becomes hyper-responsive leading to a chronic stress cortisol-laden Arndt-Schulz response and allostatic overload. This overburdens the mPFC and hippocampus, causing structural and functional degradation that handicaps cognitive flexibility [111, 112].

The end result will be gauged in the crucial capacity to regulate emotion and thereby modulate the stress response and its effects on the inflammasome and ISR. During the hormetic normal-tolerable stress phase, synaptic functioning is actually enhanced. Synaptic transmission improves, the dendritic arbor is nourished and long-term potentiation (LTP) is strengthened. Learning is supported in such a milieu and mPFC-ACC decision-making is enhanced. In the dlPFC, levels of both DA and NE will promote an inverted U-shaped curve response in terms of working memory function and in terms of dlPFC’s capacity to contribute to emotion regulation through effects on subgenual Brodmann area (BA) 25 in the ACC [74].

In the case of toxic stress, negative remodeling will suppress synaptic functioning with reductions in synaptic transmission, LTP and learning with disturbances in decision-making. Neurogenesis in the hippocampus will suffer and dendritic atrophy will be seen affecting adaptive plasticity. Structural resilience will also suffer due to impairments in remodeling and neurotransmitter distortions with delays in recovery from insults [110, 112]. Much of this may be tied to deficits in the adaptive plasticity of neurogenesis and dendritic remodeling. This lowers the threshold for future excitotoxic events.

Neuronal plasticity also appears to be subject to an inverted U-shaped curve response. Both hypoplasticity and hyperplasticity in the brain are suboptimal and can diminish overall cognitive function and brain health [113].

When one examines neuroimaging changes that accompany severe stress conditions, a sobering realization emerges. There is a clear interaction of greater subjective chronic separation stress and greater cumulative adverse life events that associate with smaller volumes in the mPFC, insula cortex (IC) and ACC region [77]. These areas are central to emotion regulation and also to attachment solution active inference decision-making.

It is fascinating to consider the spatiotemporal inter-relationship of the inverted U-shaped curves that describe the biphasic effect of separation stress in increasing amounts and frequency on intracellular organelle niches at the level of mitochondria and ER and at the next hierarchical regime level of the cell itself, in this case the neuron, but also likely at work in glial cells as well, and upstream at the level of the organism’s overall structure-function landscape.

## Decision-making and uncertainty

The brain evolved in large part as a specialized organ that executes the 4 operations of any living entity. It *senses* its environment, *analyzes* the incoming information using active inference and predictive error in order to *effect* a *motor response* when its motivational analysis warrants it [81].

In order to make the decision to avoid or approach something, we need to know *where* that something is with regard to us and also *what* it is. These questions are processed through the confluence of 2 ancient brain moieties [114]. The dorsal “where” *hippocampocentric* pathway and the ventral “what” *olfactocentric* pathway converge primarily in the ACC in the paralimbic mPFC. The ACC is charged with making response selections with the assistance of the dlPFC and the OFC to avoid or approach, separate or attach. It does so after considering *“where”* and “*what”* information along with physical and social pain signals and stored memory to which it has access. The ACC is also home to the mammalian behavioral triad of maternal nurturance, the infant’s separation cry, and social play [17]. These factors play an outsized role in titrating our motivational states because of the huge premium natural selection has placed on social attachment behavior in mammals, primates and especially on altricial human beings.

This reality is reflected in the central role the ACC and the MCC play in the CSTC Brain Reward-Motivation Circuitry. The ACC has a primary mission of motivation with energizing of action selection and the MCC has a primary role of cognitive control and hierarchical reinforcement learning (HRL) [115]. Together these regions select and maintain behavioral repertoires called *policies*. These policies are setting-related sequences that are goal directed and learned through the process of HRL [115]. In other words, the ACC/MCC selects and maintains coherent actions in the service of successful avoidance and approach.

This particular ACC/MCC CSTC circuit is at work in mediating 4 basic attachments, namely: 1) Metabolic energy source-food, water (living entities) 2) Sexual Objects (vertebrates) 3) Parental and Social Objects (primarily mammals and birds) 4) Future Objects (humans, and to a lesser extent primates and perhaps cetaceans and elephants).

Evolutionary (phylogenetic) stress and developmental (ontogenetic) stress both involve separation challenges to these 4 attachments. In these circumstances there is emergent probability that natural selection rewarded individuals and groups who had a variation that promotes an attachment solution to these challenges. In this context, stress can be understood as what the brain does to itself and the rest of the body when faced with a separation threat or challenge endangering one’s attachments. It is the threat stress of separation from any one of these 4 attachments that activates the amygdala in a turbulence of attachment uncertainty and can lead to the stress response and neurogenic neuroinflammation with eventual intolerance of this uncertainty and expulsion from the hormetic stress zone into the toxic stress zone. Resilience in essence involves a bolstering of the mPFC’s capacity to emotionally regulate amygdalar activation in the face of anomalies and uncertainties that portend the pain of separation.

It is here that the alacrity of one’s ACC/MCC in making good response selections that maintain health promoting attachments comes into play. And once again, based on stress-related effects at the mitochondrial, ER and cellular levels, we will see a self-similar biphasic inverted U-shaped curve illustrating that, in a nested fashion, hormetic stress levels will aid fitness, performance and functioning but high uncertainty from psychosocial toxic stress can lead to a pathogenic reduction in fitness and functioning.

**Fig. 9:**
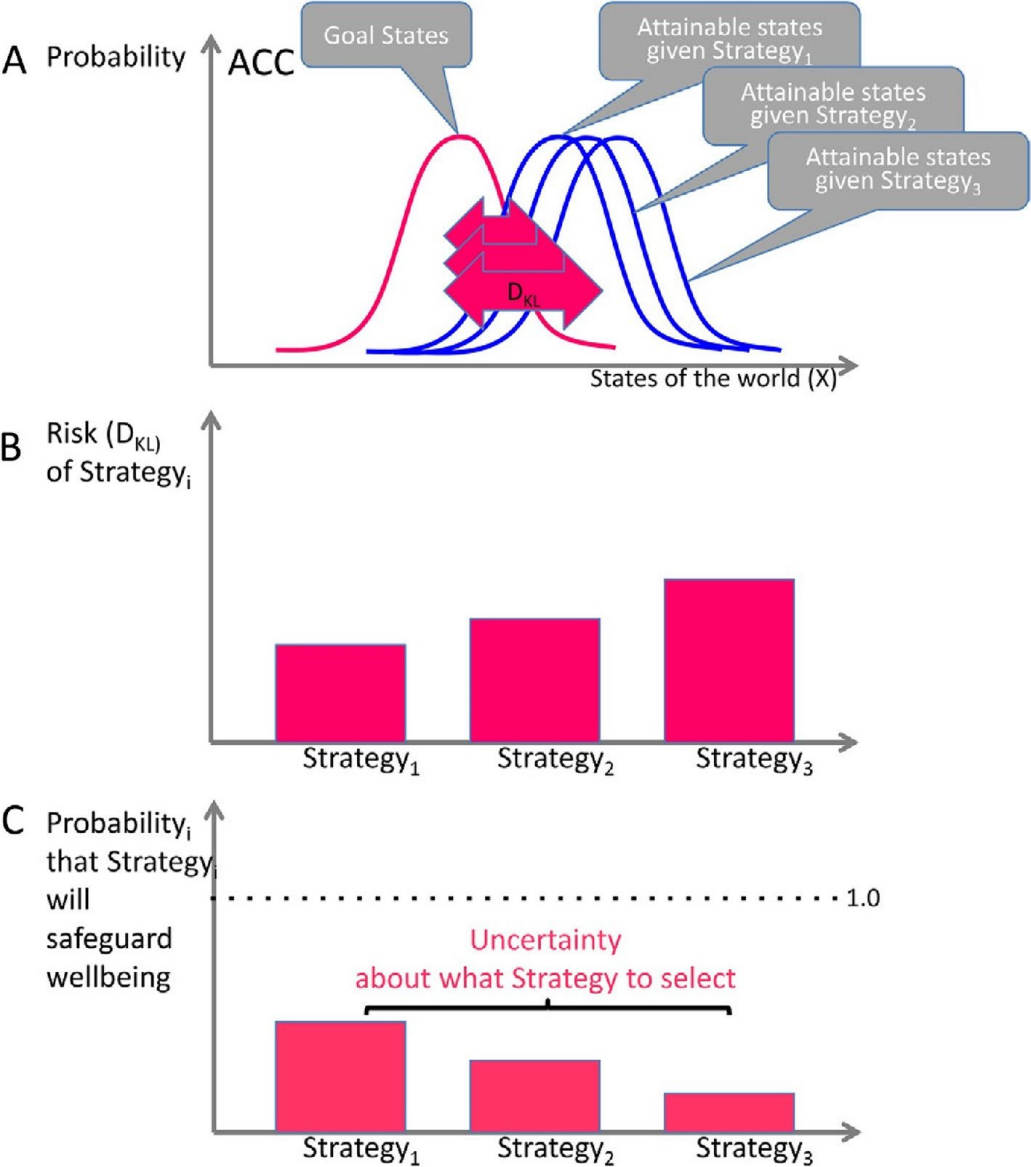
The ACC and its analyzer-effector function in selecting the best strategy. **A** During stress, the ACC assesses the gap between the probability distributions of ‘attainable states’ and ‘goal states’ in a search for plausible strategies or *“policies”* that constitute a menu of options. Such a gap or divergence is called a Kullback-Leibler divergence (DKL). The greater the relative risk represented by the divergence, the greater the uncertainty about which policy to choose leading to greater amygdalar activation. **B** Each policy represents a different relative risk (DKL). **C** Based upon the risk probability distribution, the ACC analyzes each strategy i (i = 1,2, …, n) for the probability that it will reduce negative prediction error and effect an attachment solution to the current separation challenge and thus ensure wellbeing. In this graphic, all probabilities are relatively low. Thus, uncertainty persists about the answer to the potential separation challenge. This can lead to a separation threat-based stress response due to a high level of uncertainty and the intolerance that ensues causing entropic strain with metabolic wear and tear in the system. From: Peters et al, 2017, p. 182 (open access) [116]. *"Reprinted from,* Progress in Neurobiology, 156*.* Peters A, McEwen BS, Friston K. Uncertainty and stress: Why it causes diseases and how it is mastered by the brain*. P. 182, 2017, under Creative Commons Attribution Non-Commercial No Derivatives license permission from Elsevier via STM guidelines*

If we focus our lens on the ACC, we appreciate the importance of the active inference and strategic prediction that takes place there as it engages in decision-making leading to the formation of a pre-motor template [116] (See Fig. 9).

With this in mind, we can understand how important the health of the paralimbic cortex is for overall health. Many insults, from periventricular white matter disease, to stroke and tumor and inflammatory disease and encephalitidies and peripheral infections affecting this region through fenestrated endothelium, to neurotransmitter derangements pursuant to psychiatric disorders, all can affect the ACC directly through lesions or indirectly through medial forebrain bundle modulatory interruptions. In these cases, one can expect the ACC/MCC is perform poorly in flexibly making useful decisions, thus failing to reduce uncertainty and perpetuating toxic stress.

A healthy ACC/MCC region, as the convergent watershed area for both hippocampocentric and olfactocentric flows of information, can use active inference and prediction error to form the best policies and make the best motor judgments to attain goal states [114]. It has also recently been proposed that the hippocampocentric moiety not only provides *“where”* information but also may afford our sense of *“when”* providing temporal structure in relationship to the outer world [117]. Having access to an accurate sense of time would enhance the spatiotemporal decision-making role of the ACC/MCC. And in order to make the best use of time as a metric, our inner time sense must synchronize with a reified outer time measurement.

Recently it has been proposed that optimal inner time to outer time synchronization is achieved with a medium speed of inner time [118]. Trouble arises with desynchronization when extremes of inner time speed emerge in the context of changes in mental status. When inner time becomes too fast as occurs in mania or too slow as occurs in depression, there is distortion of the match with the outer time metric. This can destabilize one of the core zeitgebers we rely on in making critical decisions regarding attainable goals. This relationship between speed of inner time on the *x*-axis and synchronization between inner and outer time on the *y*-axis forms another inverted U-shape curve in the nested hierarchy [118].

 Decision making in the ACC/MCC will often rely on working memory functioning in other parts of the PFC like the dlPFC. It is known that stress impairs working memory via high levels of dopamine D1 receptor (D1R) activation of cyclic adenosine monophosphate signaling, which reduces PFC neuronal firing [119]. This appears to happen when excessive stress increases hyperpolarization of cyclic nucleotide-gated (HCN) cation channels on dendritic spines where PFC pyramidal neurons form their cortical-cortical interconnections. This effect is also subject to a biphasic response curve. It has been shown that either too little D1R stimulation or too much will result in an inverted U dose-response effect on working memory thereby impairing cognitive functioning and handicapping ACC/MCC decision-making [120].

Northoff and Tumati (2019) highlight, in an elegant synthesis, the self-similarity of neural and mental states as reflected in the concept of the biphasic non-linear inverted U-shaped curve, which depicts the average optimal healthy state along with pathological extreme states [121]. Slow, optimal and rapid neural frequencies exist along a continuum associated with the balancing of excitatory: inhibitory circuit activity. When neural frequencies are not in a *“Goldilocks zone”* so to speak, namely too slow or too fast, they may project out to the dysfunctionality of mental features we see in neuropsychiatric disorders. These authors call this process a neural-mental transformation according to the spatio-temporal dynamics of neural activity, which are bound to a non-linear scalar rule along with the multiple other examples presented in this paper [79, 80].

The spatio-temporal dynamic view of neural-mental transformation can be subsumed in the inverted U-shaped nature of non-linear scalar self-similarity at each system level in the nested hierarchical organism.

## Enhancing and extending biological performance, health and resilience

Biological fitness appears to improve as a function of repeated exposures to optimized hormetic stress stimuli applied at rhythmic intervals [33]. A single hormetic stimulus will produce a time-limited modest improvement somewhere in the range of a 30-60% improvement to the genetically determined peak of an individual. Repeated exposures strengthen, in stepwise way, the layers of a strong resilience for a lasting, saludogenic response.

Mind body interventions (MBIs) may be able to enhance and extend resilience through hormetic processes. This possibility can be explained through an understanding of stress-related gene expression and immunoactivation and an examination of how a person can impact gene activation states through behavioral change. Buric and colleagues (2017) reviewed clinical and non-clinical studies using a variety of research designs that have studied gene expression analysis in relaxation response eliciting MBIs (i.e., mindfulness and other meditation techniques, yoga, Tai Chi, Qigong, and breath regulation etc.) [122]. Eighteen relevant studies were analyzed. Overall, the studies indicate that MBIs are associated with a downregulation of the NF-κB gene ontology pathway. Thus, it appears that MBIs may attenuate the adverse impact that chronic stress has on pathogenic IRS and ISR-related gene expression, with implications for health promotion and illness prevention, notably among individuals with heightened chronic stress, including marginalized racial or ethnic groups or individuals of low SES.

Population based scalable MBIs designed to mitigate stress-related risk and promote resilience or recovery in high-risk populations, when combined with structural community-based interventions, could help improve our long-term response to PAMP and non-PAMP crises and help prepare at-risk communities more effectively for future public health emergencies. The suboptimal response to the COVID-19 pandemic is certainly a cautionary case in point [123]. Behavioral and MBI approaches can help manipulate the bidirectional CNS–immune regulatory system by affecting gene expression changes in stress and the immune response.

This integration of MBIs and lifestyle change may enhance the amplitude of the hormetic maximum an individual can muster in the face of intermittent, repeated mild to moderate stressors setting the mind body medicine stress to resilience ratio to a healthy stress rejuvenescent level [124].

**Fig. 10:**
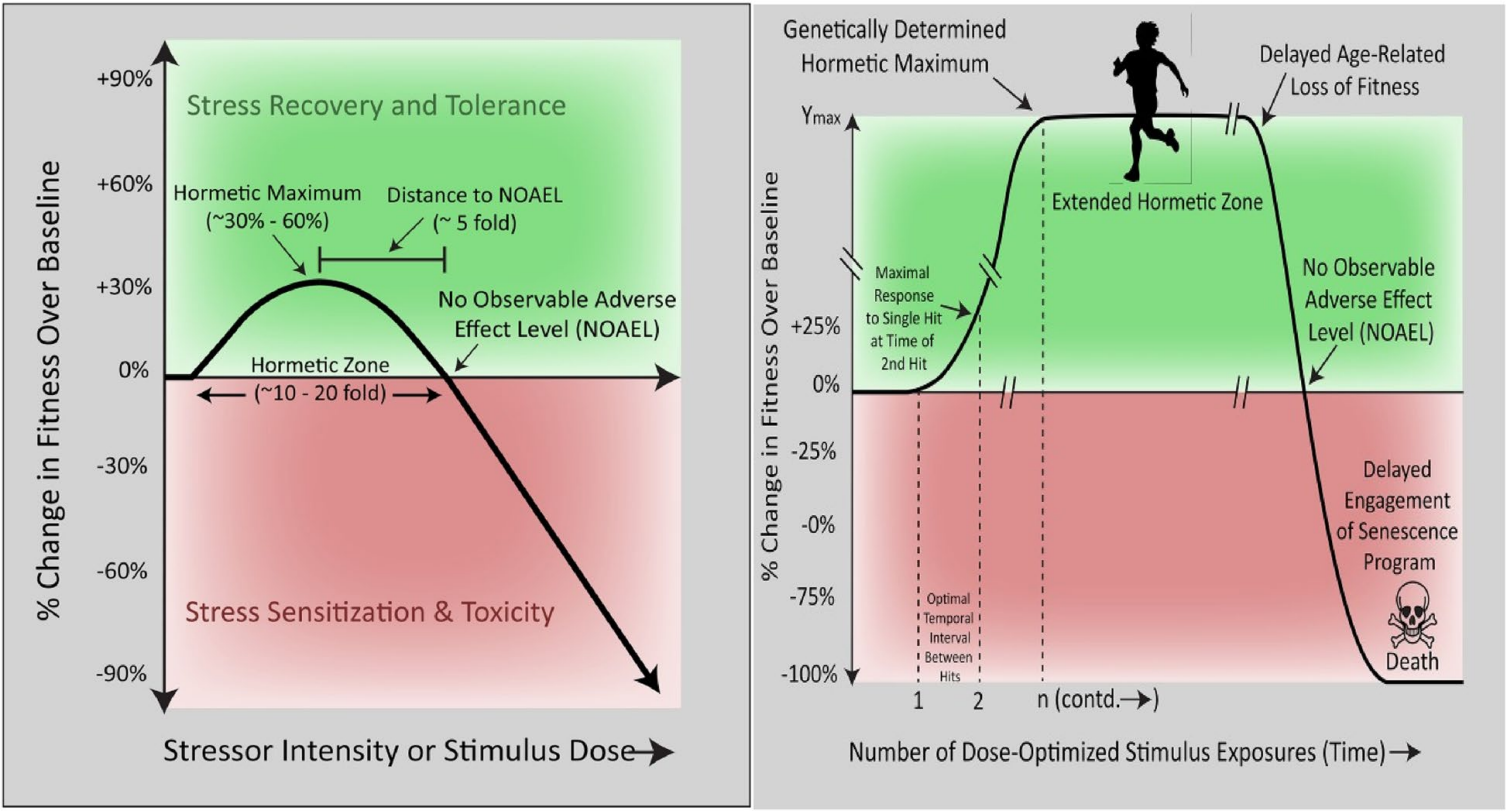
Enhancing biological performance and resilience through the extension of the Hormetic Zone is a potential niche for mind body therapies to exploit. One hormetic stress response will result in a modest amplitude effect and a time-limited response. When hormetic stress responses, perhaps in the form of mind body practices, are performed repeatedly, these responses may summate in stepwise manner and may extend the hormetic zone. From: Leak et al, 2018, [124]. Reprinted from: Dose Response. 2018; 16(3). Leak, R. K., Calabrese, E. J., Kozumbo, W. J., Gidday, J. M., Johnson, T. E., Mitchell, J. R., Ozaki, C. K., Wetzker, R., Bast, A., Belz, R. G., Bøtker, H. E., Koch, S., Mattson, M. P., Simon, R. P., Jirtle, R. L., & Andersen, M. E. Enhancing and extending biological performance and resilience, with permission from Sage Publications

This offers a foothold for effective health promotion and primary, secondary and tertiary disease prevention. Eventually there may come a day when polygenic risk factor analysis for certain stress-related NCDs will prompt the use of MBIs as part of a personalized secondary prevention strategy [125]. Environmental stimuli such as mind body activity/dietary/lifestyle factors can be optimized so that the maximal epigenetic/genetic potential can be reached [124, 125] (See Fig. 10). As referred to above, there is early suggestion that relaxation response eliciting MBIs, which can impact the biological, psychological and social vectors of health and illness, may be somewhat effective in reducing the accelerated aging picked up in DNAm epigenetic clocks [70]. The yet to be determined relationship between epigenetic clocks and the maintenance of the hormetic stress zone invite further research and may hold a key to the biopsychosocial nature of medicine.

## The inverted U-shaped curve, allometric scaling laws and fractal self-similarity

This paper has featured the similarities that abound along the multiple stratifications that make up the nested hierarchy of the human organism. These self-similarities are depicted in the standard Gaussian inverted U-shaped curve approximations depicted in the figures above. The *Bell-shaped cu*r*ve* describes the statistical variation around an average value of some sort and so is frequently seen across scientific disciplines.

But certain refinements are needed to better understand these relationships [79]. The brain and its neural networks have evolved to, on average, use the minimal amount of energy in the performance of the everyday living needed for self-preservation. By doing so, *free energy minimization* is achieved, which in turn maximizes the amount of energy available for species preservation, namely sexual activity, reproduction and care of offspring. Health lies in the balance since as we grow and age, the amount of energy we require simply for cell maintenance, based on a linear increase, will double (2x) as our cell number doubles, while our supply of energy based on metabolic rate will only increase sub-linearly as a ¾ power exponent (1.682x) [79]. As stated earlier, the rate at which energy is needed for maintenance, will eventually outpace the capacity of the organism to produce energy and, especially if maintenance is strained by toxic stress, this will result in allostatic overload.

This relates to the aforementioned *Kleiber’s Law* [79]. A straight line on a logarithmic plot, with increase by factors of 10 on both axes, represents a sub-linear scaling power law with its exponent reflecting the line’s slope. For every 4 orders of magnitude we see with increase in mass plotted on the *x*-axis, we will see an increase of only 3 orders of magnitude in metabolic rate on the *y*-axis. This results in a slope with the Kleiber’s law exponent of ¾.

As an organism grows it not only needs energy to make new cells, it needs it to maintain old ones. This means that the metabolic rate available to create new cells, is the quantity of metabolic energy produced by mitochondria left after that used for the maintenance of existing tissue [79]. Consider the maintenance energy needs when an individual is under great levels of toxic stress producing allostatic overload. As it increases by say 100%, the metabolic energy supply will be incapable of keeping up. It will only be capable of increasing at 75%. This constitutes the kind of MetS deficit spending that will eventually cause tissue damage compounded by reduced creation of new cells. And here is where the concept of vulnerability to illness and accelerated biological aging secondary to allostatic overload comes into focus, providing a scientific infrastructure for biopsychosocial mind body medicine.

This is summarized in an extraordinarily important *allometric* (meant to represent different sizes and shapes) *scaling law*, namely that the metabolic rate, which is the rate at which energy is supplied to cells, is the sine qua non underlying all other biological rates. We are presented with a single universal parameter, which is the average activation energy required to produce a single molecule of ATP during mitochondrial oxidative metabolism, namely approximately 0.65 electron volts (eV) [79, 80]. If we then start with mitochondria, we can see the inverted U-shaped curve that describes the mitochondrion’s response to Kleiber’s Law. Low level hormetic stressors will improve mitochondrial function making optimal use of metabolic rate with the production of ATP for cellular use both for self-maintenance and species preservative functions. When there is chronic or overwhelming toxic stress, the ¾ magnitude increase in metabolic rate is outstripped by maintenance costs leading to the pathogenesis of allostatic overload and accelerated biological aging [79, 80].

This is recapitulated in the iterative phenomenon called *fractal self-similarity* reflected in the metaphor of the Russian dolls. Each level in the nested hierarchy in essence replicates a scaled version of the adjacent level. In the context of the inverted U-shaped curve, each level from mitochondria to ER to cell to brain as organ to organism to community of organisms will exemplify self-similarity in the effects of stress experienced (normal, tolerable and toxic) and the stress resilience enjoyed at each particular systems level. Vulnerability to stress-related illness will emerge when stress becomes toxic and/or resilience sags. This bidirectional process can work its way up in patients who have mitochondrial dysfunction with mito-allostatic overload for example, or it can work its way down in patients who have psychosocial stress with allostatic overload. All systems in the hierarchy can thus wind up with a pathogenic curve.

It should be noted that the Gaussian Bell-shaped curve is best reserved for entities that are characterized by independent, random, and uncorrelated variables. In living organisms there may be many more rare events (*“Black Swans”*) than would be expected from the randomness of independent variables described by the normal Gaussian distribution [126]. The tendency to see correlations in living organisms may lead to a non-randomness better characterized by graphs with so-called *“fat tails”*. These non-linearities in biology lead to hormesis graphs marked by a *“dose response”* that is convex (antifragile) representing a sloping increase in effectiveness at tolerable levels of stress. Additional “doses” of stress tend to be less and less effective until they actually become “concave” (fragile), representing harm.

The issue of *“fat tails”* is tied up with the problem of uncertainty, when random events emerge outside the confines of standard deviation. The more uncertainty one finds in a model constructed to calculate probabilities, the more underestimation there will be small probabilities that will reside in the fat tail regions of the curve [126]. In other words, when information flows in from an organism's non-random association-related fat-tailed distribution, relying on a normal distribution curve might underestimate true predictive risk. Here we may infer a mathematical infrastructure for the human problem we call *intolerance of uncertainty,* which is responsible for the transdiagnostic problem we often see in emotion regulation when fear of anomalies that may suggest a separation challenge, with its attendant heightened amygdalar tone, overcomes the capacity of the mPFC to dampen it leading to toxic stress.

So, we may speculate that many of the fractally self-similar inverted U-shaped curves included in this article may be consonant with power laws that obey non-linear hormetic stress power law dynamics. Clearly more research in biopsychosocial mathematics will be needed to better understand this hierarchical network complexity.

### Summary

In this speculative review paper based on the recent literature, we have contended that toxic separation stress, heightened by insecure attachment, low socioeconomic status and social isolation stress, will over time predilect to premature illness and accelerated aging.

This is because toxic stress creates overwhelming maintenance energy needs and often eventuates in allostatic overload.

Each layer in the nested hierarchy is affected giving rise to self-similar inverted U-shaped curves up and down the hierarchy contributing to the organism’s holistic performance, health and aging curves.

Active inference under hormetic stress resilient conditions will confer free energy minimization in the ACC paralimbic cortex in essence providing a metabolic energy savings account. This can be applied to second (sexual object attachment), third (parental and social attachment) and fourth (future object goal attachment) order attachment needs.

Thwarted attachments create separation stress at each of the 4 attachment levels. This can bump tolerable hormetic stress into the toxic stress zone maximizing metabolic rate strain and making allostatic overload more prominent leading to MetS and changing what may have once been a virtuous cycle into a vicious one.

In immune cells under these circumstances, excessive stress-related DAMPS will upregulate NF-κB pathways as hypothesized in the concept of the conserved transcriptional response to adversity (CTRA), with potential implications not only for stress-related NCDs but for viral illnesses as well [127, 128]. When mired in this chronic innate pro-inflammatory state, there will be increasing vulnerability to stress acceleration and allostatic loading that pushes the individual outside the hormetic stress zone and into toxic stress setting the stage for stress-related NCDs and viral illnesses (e.g., coronaviruses) as well as to *inflammaging* with accelerated biological aging [34, 128].

Mind body approaches, representing attachment solutions to life’s separation challenges, join hormetic stress (mild intermittent tolerable separation stress) and healthy lifestyle approaches (sleep hygiene, exercise and low glycemic diet) in enhancing stress resilience and stress rejuvenescence. A beneficial stress to resilience mind body ratio may result leading to enhancement of biopsychosocial performance and resilience through the extension of the hormetic zone. At the level of the mitochondria, this so-called *protective embedding* can enhance the pro-longevity hormetic zone in the biphasic inverted U curve by reducing expression of NF-κB pathway gene expression thus dampening oxidative stress, the IRS and *inflammaging.* This may strengthen mitochondrial reserve capacity and reduce mito-allostatic loading and the need for the ISR and subsequent apoptosis, thus favoring beneficial cellular health. The fact that several MBIs have been found to reduce gene expression of the NF-κB gene ontology set makes them potentially important adjuncts in a pro-active approach to health promotion [122].

According to nested hierarchical theory, these inter-relationships will also enhance other *“protective embedding”* effects in inverted U-shaped systems along the organism’s complexity continuum that rely on hormetic subsystems. When the stress to resilience ratio in sub-systems is maintained in the hormetic zone, neuronal synaptic structure and function and the ACC inferential decision-making capacity will improve through the finding and successful execution of attachment solutions (food, sex, social and future object attachments). This stress resilience strengthening will lead to the reduction of allostatic loading and the advancement of health and performance.

Engel’s intuition regarding the biopsychosocial nature of medicine predated the recent revolution in our understanding of epigenetics and the pro-inflammatory pathogenic stimulation in brain and body that accompanies toxic separation stress. The epigenetic revolution in medicine provides a scientific rationale to help us understand the hormetic and allostatic forces at work in the biological, psychological and social matrix that forms the human experience of health and illness.

We may surmise that stress will impact a nested hierarchy of biological inverted U-shaped curves (e.g., mitohormesis, proteostasis, neuronal synaptic functioning, ACC Bayesian active inference curves) and consequently and bidirectionally, psychological bi-phasic inverted U-shaped curves (e.g., Yerkes-Dodson curve). One might even speculate on the existence of societal inverted U-shaped curves (e.g., Stimson economic, political, social and cultural curves).

**Fig. 11:**
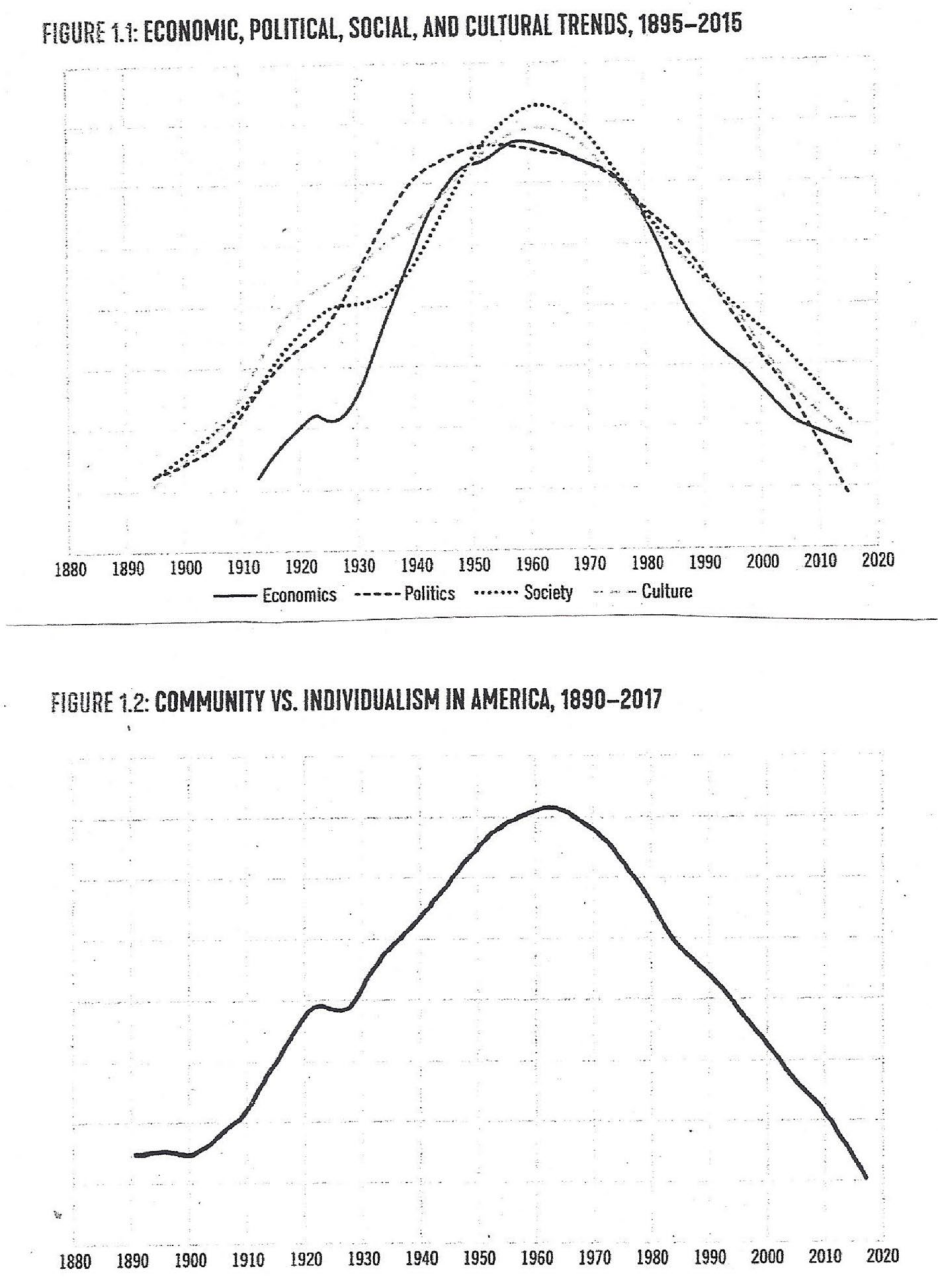
A Nested Hierarchy of Inverted U-shaped Curves. Individual curves, in social network theory, interact and summate locally and nationally to contribute to economic, political, social and cultural Bell-shaped curves. From: Putnam and Garrett, 2020 [129]*. “Reprinted from,* Putnam R, Garrett SR. The Upswing. How America Came Together a Century Ago and How We can Do It Again. NY: Simon and Schuster, 2020, *… with permission from Simon and Schuster"*

Putnam and Garrett, (2020) do just that in their recent book, *“The Upswing. How America Came Together a Century Ago and How We can Do It Again”,* using a methodology called The Dyad Ratios Algorithm devised by Stimson to graph economic, political, social and cultural trends against the years from 1880 to 2020 [129, 130]. These individual curves, in social network theory, will interact and summate locally and nationally to contribute to economic, political, social and cultural Bell-shaped curves (See Fig. 11).

When these 4 curves are blended using a summary graph based on separate Stimson type factor analyses combining all variables from all of the 4 domains, we find another Bell-shaped curve [129, 130]. This summary graph suggests that the single most important relationship responsible for the inverted U-shaped summary curve with its peak around 1960 for all 4 variables was the strengthening of communitarian values and interest in policies that emphasized the commonweal. The Progressive Era *“upswing period”* lasted from the turn of the 20^th^ century up to around 1960 as opposed to the narcissism and individualism that preceded it in the Gilded Age and appears to have followed it in present day America. There have been separative challenges at work in economics (the wealth gap), politics (polarization), social life (isolation and loneliness), and culture (narcissism). This contrasts with the Progressive Era which featured clear-cut attachment solutions to the separation challenges of society. The Progressive era’s *“...legacy is nonetheless clear—the hard measures of economic equality, political comity, social cohesion, and cultural altruism.”* [129] (Putnam and Garrett, p.339).

Interesting new research in the social transmission of stress in animal collectives may offer us a framework for understanding this cumulative societal inverted U-shaped curve [131]. There is now sufficient evidence to suggest that stress transmission is widespread across vertebrates and this may be especially true among mammals given our social attachment survival strategy. Most of this work has involved dyadic stress transmission, but there is evidence that stress transmission could be occurring on larger scales that could include economic, political, social and cultural separation challenges. According to social network theory, stress transmission processes can spread this contagion in social groups contributing the emergence of widespread neurobehavioral features. This effect can include the attachment solution of cooperation in human communities [132]. However, more research on stress transmission in animal and human social systems will be required to elucidate the stress transmission process and its possible mechanisms and consequences.

One can speculate that the concept of attachment solutions to separation challenges lies at the core of each individual’s evolutionary separation stress to attachment-based resilience ratio as depicted in the nested hierarchy of biopsychosocial inverted U-shaped curves [27]. The hypothesis suggests that each inverted U-shaped curve bidirectionally impacts the next level of hormetic stress in each person. The nature of non-linear scalar self-similarity at each hierarchical level is occurring in differential moment to moment time frames synthesized according to the evolutionary reckoning imposed by separation challenges that call for attachment solutions.

The Stimson-type factor analytic synthesis of economic, political, social and cultural curves is possibly just a population-based macrocosmic version of the naturally selected mammalian survival strategy of meeting separation challenges with attachment solutions. This separation-attachment interrelationship can be conceptualized in a hormetic inverted U-shaped curve with separation stress plotted on the *x*-axis and attachment solutions on the *y*-axis. And when we derive the Mind Body Medicine separation stress to resilience ratio, we can speculate on the wisdom of finding cooperative ways to maintain our attachments to metabolic energy, sexual partners, parental and social objects and future goals. This, we may surmise, promotes the health and the flourishing of both individuals and communities.

We arrive back at the core separation challenges to the 4 evolution-based attachments of human existence that form the basis for biopsychosocial medicine. Could it be that when these basic separation stressors are extrapolated as societal challenges and are balanced by the social resilience and collective efficacy that comes with communitarian attachment solutions, we stay as a society on the upswing hormetic side of the biphasic inverted U-shaped Stimson summary graph? If true, this kind of healthy community will promote the health of those living in the community just as these healthy, securely attached individuals will contribute to the healthy community itself.

Modern mind body medicine offers us a scientific narrative with a way to understand the interplay of biological and psychological and social factors and to integrate these vectors in whole person care at the bedside and in the clinic. Moreover, it supplies us with a unified approach to population health promotion and primary, secondary and tertiary prevention allowing us to envision a time when there will be societal support for a population-based approach that links clinical medicine with public health [133].
